# Single‐cell RNA‐seq analysis reveals the platinum resistance gene COX7B and the surrogate marker CD63

**DOI:** 10.1002/cam4.1828

**Published:** 2018-10-26

**Authors:** Nobuyuki Tanaka, Shintaro Katayama, Aparna Reddy, Kaneyasu Nishimura, Naoya Niwa, Hiroshi Hongo, Koichiro Ogihara, Takeo Kosaka, Ryuichi Mizuno, Eiji Kikuchi, Shuji Mikami, Ayako Miyakawa, Ernest Arenas, Juha Kere, Mototsugu Oya, Per Uhlén

**Affiliations:** ^1^ Department of Medical Biochemistry and Biophysics Karolinska Institutet Stockholm Sweden; ^2^ Department of Urology Keio University School of Medicine Tokyo Japan; ^3^ Department of Biosciences and Nutrition Karolinska Institutet Huddinge Sweden; ^4^ Department of Diagnostic Pathology Keio University Hospital Tokyo Japan; ^5^ Department of Urology Karolinska University Hospital Solna Sweden; ^6^ Department of Medical and Molecular Genetics King’s College London London UK; ^7^ Keio University Graduate School of Medicine Tokyo Japan

**Keywords:** CD63, COX7B, platinum resistance, single‐cell RNA‐seq, tumor heterogeneity

## Abstract

Cancers acquire resistance to systemic treatment with platinum‐based chemotherapy (eg, cisplatin [CDDP]) as a result of a dynamic intratumoral heterogeneity (ITH) and clonal repopulation. However, little is known about the influence of chemotherapy on ITH at the single‐cell level. Here, mapping the transcriptome of cancers treated with CDDP by scRNA‐seq, we uncovered a novel gene, *COX7B*, associated with platinum‐resistance, and surrogate marker, CD63. Knockdown of *COX7B* in cancer cells decreased the sensitivity of CDDP whereas overexpression recovered the sensitivity of CDDP. Low *COX7B* levels correlated with higher mortality rates in patients with various types of cancer and were significantly associated with poor response to chemotherapy in urinary bladder cancer. Tumor samples from patients, who underwent CDDP therapy, showed decreased COX7B protein levels after the treatment. Analyzing scRNA‐seq data from platinum‐naïve cancer cells demonstrated a low‐*COX7B* subclone that could be sorted out from bulk cancer cells by assaying CD63. This low‐*COX7B* subclone behaved as cells with acquired platinum‐resistance when challenged to CDDP. Our results offer a new transcriptome landscape of platinum‐resistance that provides valuable insights into chemosensitivity and drug resistance in cancers, and we identify a novel platinum resistance gene, *COX7B*, and a surrogate marker, CD63.

## 1 INTRODUCTION

Cancers are assemblies of heterogeneous cell populations of various sizes, different genetic compositions, and distinct phenotypic characteristics.^1^, ^2^, ^3^, ^4^, ^5^, ^6^, ^7^, ^8^, ^9^ This so‐called intratumoral heterogeneity (ITH) is central for the natural selection that drives carcinogenesis and tumor development.^1^, ^3^, ^6^, ^9^, ^10^, ^11^, ^12^ Recent technical advances in genome sequencing have been elucidated in a model of Darwinian selection, in which cancer cells develop with repeating oncogenic mutations, resulting in cellular clonal expansion and extensive ITH.^3^, ^13^, ^14^ Clinically, the relationship between ITH and the efficacy of cancer therapy is evident.^7^, ^11^, ^14^, ^15^, ^16^ For example, the linker histone H1.0 has been shown to exhibit high inter‐ and intratumor heterogeneity in numerous cancer types, with H1.0 levels correlating with tumor differentiation status, patient survival, and, at the single‐cell level, cancer stem cell markers.^7^ In patients with prostate cancer, analysis of circulating cell‐free DNA could identify multiclonal heterogeneity of *BRCA2* reversion mutations that was associated with resistance to PARP inhibitors (Olaparib, US brand name Lynparza).^17^ Platinum‐based cisplatin (CDDP, US brand names Platinol, Platinol‐AQ) is one of the most effective chemotherapy agents for many types of cancers. However, CDDP treatment often causes phenotypic alterations to the original tumor.^18^, ^19^, ^20^ We hypothesize that CDDP induces a rather drastic change in the ITH at a single‐cell level, eventually leading to the development of acquired resistance to CDDP.^21^ Until recently, little has been known about ITH states before and after platinum treatment. Such knowledge could be essential to understanding the mechanisms leading to platinum‐resistance.^22^, ^23^

To examine ITH states before and after platinum treatment, we applied the latest technology of single‐cell RNA‐seq (scRNA‐seq). The scRNA‐seq system has been developed to investigate cellular heterogeneity, uncovering new cell types and sub‐populations.^24^, ^25^, ^26^, ^27^, ^28^ In malignancy, this high‐end technology enables us to scrutinize ITH in bulk cancer cells.^2^, ^5^, ^6^, ^8^, ^9^, ^29^ Studying urinary bladder cancers at the single‐cell level, we first revealed a dynamic shift in the heterogeneity of cancers following treatment with CDDP. Second, we identified a novel gene, *COX7B,* associated with platinum‐resistance. Third, we demonstrated a low *COX7B* subclone, behaving as cancer cells with acquired platinum‐resistance in platinum‐naïve cancer. Forth, we reveal a surrogate marker, *CD63* that can distinguish low *COX7B* subclones. These results offer further platinum‐resistance knowledge that can be used for future clinic diagnosis.

## 2 METHODS

### 2.1 Single‐cell preparation, isolation, and cDNA synthesis

The cultured cells were suspended in a trypsin solution and centrifuged at 150 *g* for 5 minutes. The cell suspension was then filtered twice through a 20‐μm strainer and maintained on ice. Prior to single‐cell isolation, the cells were photographed for viability and cell size measurement using the EVE Automated Cell Counter (NanoEnTek Inc., Seoul, South Korea). Viability was measured using trypan blue exclusion, which confirmed >90% cell viability. The mean values of the measured cell sizes are indicated in Figure S1A. Next, single cells were isolated at 4°C and processed on a Fluidigm C1 platform.^24^, ^30^ Briefly, the floated cells were captured on a medium microfluidic C1 chip (designed for 10‐17 μm cells) and seeded in the wells of a 96‐well plate containing C1 Suspension Reagent. The capturing efficiency was evaluated using a Nikon TE2000E automated microscope, and a bright‐field image of every capturing position was obtained at 20× magnification using μManager software (https://www.micro-manager.org). Finally, each capture site was manually inspected for quality control and only capture sites containing single, healthy cells were further processed. Following image acquisition, RT and PCR mix was added for cDNA synthesis.^24^, ^30^ The harvested cDNA quality was measured using an Agilent BioAnalyzer.

### 2.2 Single‐cell RNA sequencing, data processing, and analysis

The STRT Seq libraries were sequenced using HiSeq 2000, and the raw sequences were preprocessed using STRTprep^31^ (commit d7efcde of https://github.com/shka/STRTprep). Briefly, the raw reads were filtered based on the quality and redundancy, and the filtered reads were aligned to the human genome hg19, the human ribosomal DNA repetitive unit (GenBank: U13369.1), the *Escherichia coli* ynbA (GenBank: EF011072 as a negative control), and the ERCC spike‐in RNAs by TopHat2.^32^ Reads within the 5′‐UTR or up to 500 bp upstream of the protein‐coding genes were counted, and the counts were divided by the total counts on the spike‐in RNAs for normalization. The distribution of the spike‐in read counts, estimated total transcript counts, and the 5′‐end capture rates were evaluated, and outlier cells on the distributions were excluded from further analysis. Significances of fluctuating (adjusted *P* value < 0.05) and differentially expressed (*q* value < 0.05) genes between cell groups were selected using SAMstrt^33^ with Benjamini‐Hochberg procedure, as described elsewhere.^31^ In brief, the technical variation was modeled based on a variation of the spike‐in RNA levels among the cells, and the significance of fluctuation in each gene was estimated by comparison between the actual variation of the gene and the expected variation based on the spike‐in. The “DE score” column in Tables S2‐S5 represents the statistical values depicting the degrees of differential expression between the represented scRNA‐seq libraries. A positive DE score represents higher mRNA levels in the latter library. A Gene Ontology term enrichment test was performed using the GOrilla web tool^34^; the ranked gene lists contained all detected genes, ordered based on the DE score.

### 2.3 Cell lines and culture conditions

Urinary urothelial cancer cell line 5637 was obtained from the American Type Culture Collection (ATCC) and was certified mycoplasma‐free. 5637PR cells were established in our laboratory as an acquired platinum‐resistant sub‐line of 5637.^21^, ^35^ Briefly, 5637 cells were passaged 1‐2 times per week in medium containing CDDP over a 6‐month period, with a gradual increase in CDDP concentration up to 3 μmol/L. Parental 5637 cells were also continuously cultured and passaged during the study period. The doubling time of 5637PR cells was slightly increased compared with parental 5637 cells, as previously described.^35^ All cells were routinely maintained in RPMI‐1640 (Invitrogen) supplemented with 10% fetal bovine serum at 37°C in a humidified 5% CO_2_ atmosphere, and current examinations were performed 3 months after ending CDDP exposure. CDDP was generously supplied by the Nippon Kayaku Co. (Tokyo, Japan) or purchased from Sigma‐Aldrich.

### 2.4 Statistical analysis

For the human studies, the samples were randomly collected with regards to the systemic chemotherapy used during 2004‐2011. Human tissue studies were deciphered with numbers to avoid investigator bias during image and data analysis, and all demonstrated at Keio University School of Medicine, Tokyo, Japan. All the experiments using human samples were ethically approved (approval # 20130095, 20130101, Keio University School of Medicine, Tokyo, Japan). No statistical method was used to predetermine sample group sizes. The values are presented as the mean ± SEM, median, and interquartile range for continuous variables, and the frequency with percentage for categorical variables. Variables between groups were compared using the two‐tailed Student's *t* test, the chi‐square test, the paired *t* test, and the Mann‐Whitney *U* test, as appropriate. Survival curves were estimated using the Kaplan‐Meier method. The indicated genes with mRNA levels of <25th percentile were considered as low. The *P* values and hazard ratios displayed on the survival plots are from the result of proportional hazards analysis using the log‐rank test. Univariate and multivariate Cox regression models with stepwise selection were used to evaluate variables for overall mortality. Spearman's and Pearson's coefficients were used to identify and evaluate the strength of the relationship between the two sets of data. To assess the ability of *CD63* to distinguish cancers with low‐*COX7B*, we performed a receiver operating characteristic (ROC) curve analysis. An area under the curve (AUC) value of 1.0 represents perfect discrimination, and a value of 0.5 represents no discrimination. Differences among groups were regarded as significant when *P < *0.05. All analyses were performed using the SPSS version 22.0 (IBM‐SPSS Inc, Tokyo, Japan) statistical software package and JMP version 13.0 (SAS Institute Inc, Cary, NC, USA).

### 2.5 Small interfering RNA, gene overexpression and transfection, cell viability assay, web‐based dataset analysis, Immunohistochemical analysis of clinical specimens, fluorescence‐activated cell sorting (FACS)

These materials and procedures are described in the Appendix S1.

## 3 RESULTS

**Figure 1:**
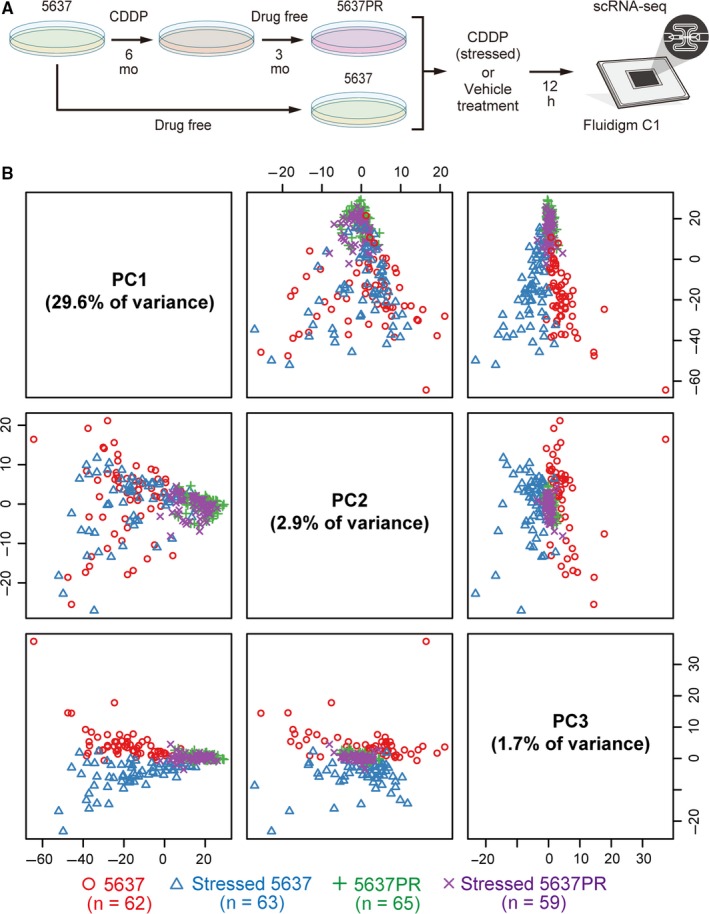
Dynamic shift of intra‐tumor heterogeneity in cancer cells assessed by scRNA‐seq. A, Workflow for studying platinum resistance in human urinary bladder cancer cells by scRNA‐seq. B, Principal component analysis (PC1 vs PC2, PC1 vs PC3, PC2 vs PC3) of the 1463 fluctuated genes in all 249 single cells (62 [5637], 63 [stressed 5637], 65 [5637PR], and 59 [stressed 5637PR]) prepared by single‐cell tagged reverse transcription

First, we designed a workflow to detect transcriptome differences between urinary bladder cancer cells that were responsive (5637) or resistant (5637PR) to platinum treatment, applying Fluidigm C1 scRNA‐seq (Figure 1A). The 5637PR subpopulation were derived from 5637 cells and had acquired platinum‐resistance.^21^, ^35^ By treating these cells with either CDDP or vehicle before sequencing^36^ we designed a model with four cell libraries: 5637 (n = 62), stressed 5637 (n = 63), 5637PR (n = 65), and stressed 5637PR (n = 59), which were prepared by single‐cell tagged reverse transcription (STRT).^24^ Interestingly, analyzing the STRT data demonstrated a significant difference between the RNA mapped/spike‐in reads among the four libraries (Figure S1B, Table S1).

In total, 1463 protein‐coding genes fluctuated (*P* < 0.05; among totally 8208 genes), and distinct ITH profiles were observed at the transcriptome level between the individual libraries (Figure 1B, Figure S1C),^33^ creating three major cell clusters (Figure S2). To achieve a scRNA‐seq fingerprint of platinum‐resistance, we next examined differentially expressed (DE; *q < *0.05) genes among the total 1463 genes identified. A total of 1132 DE genes for 5637 vs 5637PR (Table S2), 334 DE genes for 5637PR vs stressed 5637PR (Table S3), and 91 DE genes for 5637 vs stressed 5637 (Table S4) were identified. Marked differences in Gene Ontology term enrichment tests were observed between parental and established lines, demonstrating that 5637PR cells were more proliferative after CDDP, in contrast to 5637 cells (Figures S3 and S4).

**Figure 2:**
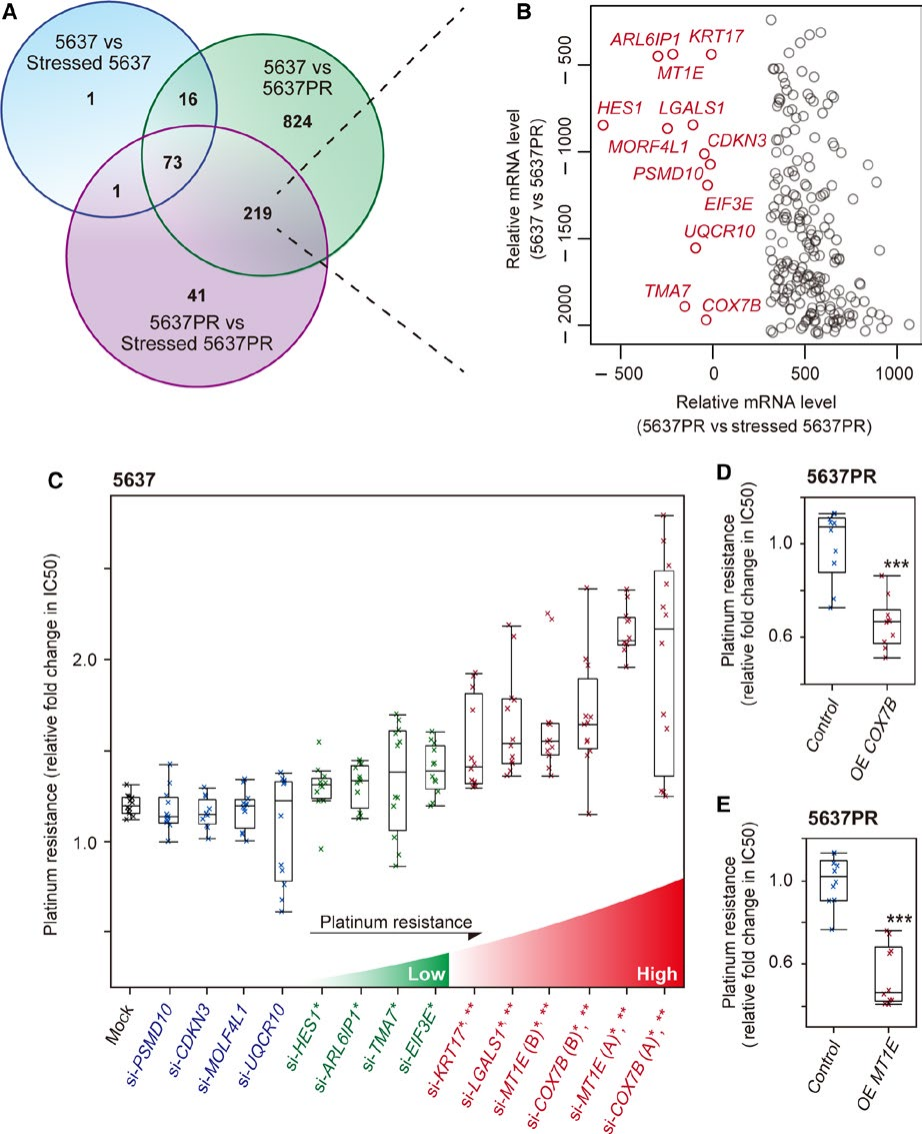
Identification of platinum‐resistance genes. A, The Venn diagram depicts the overlap between differently expressed (DE) genes for 5637 vs 5637PR, 5637PR vs stressed 5637PR, and 5637 vs stressed 5637. B, Scatter plots showing a time‐course change in the mRNA levels of the 219 DE genes between 5637, 5637PR, and stressed 5637PR cells. C, Effect of CDDP on the viability of 5637 cells transfected with siRNAs against indicated genes measured as relative fold change in IC50 (the half maximal inhibitory concentration). Mock‐transfected cells were used as control. *P < 0.05, compared with mock‐transfected cells; **P < 0.05, compared with platinum‐resistant *HES1* gene knockdown cells. D,E, Effect of CDDP on the viability of 5637PR cells overexpressing indicated genes measured as relative fold change in IC50. OE, over expressed. ***P < 0.05, compared with control cells. The *P* value from the two‐tailed Student's *t* test. The line within the box represents the median. The upper and lower quartiles are the bounds of the box, and the minimum and maximum values are the bars

Analyzing the DE genes with a Venn diagram revealed the overlapping genes between the three combinations (Figure 2A). We focused on the 219 DE genes that were changed during stages of 5637 vs 5637PR and 5637PR vs stressed 5637PR, since the DE genes for 5637 vs stressed 5637 did not associate with developed platinum‐resistance (Figure 2A). Among these 219 genes, the patterns of changes in gene expression varied (Table S5); however, 12 genes (5.5%; *COX7B*,* MT1E*,* LGALS1*,* KRT17*,* EIF3E*,* TMA7*,* ARL6IP1*,* HES1*,* UQCR10*,* MORF4L1*,* CDKN3*, and *PSMD10*) were consistently down‐regulated in platinum‐resistant cells (Figure 2B, Figure S5). Remarkably, none of the 219 genes were consistently up‐regulated. The 12 consistently down‐regulated genes were further studied, under the assumption that they were crucial for platinum‐resistance.

Then, we transfected siRNAs against the 12 candidate platinum‐resistance genes to platinum‐naïve parent 5637 cells and examined their sensitivity to CDDP by assessing cell viability (Figure 2C). The results revealed that among the 12 genes knocked down, eight genes (*COX7B*,* MT1E*,* LGALS1*,* KRT17*,* EIF3E*,* TMA7*,* ARL6IP1*, and *HES1*) significantly decreased the sensitivity to CDDP compared to mock‐transfected cells. Additionally, there was a difference in the impact on platinum‐resistance for four genes (*COX7B*,* MT1E*,* LGALS1*, and *KRT17*) having significantly higher platinum‐resistance (Figure 2C). To investigate whether we could rescue the sensitivity of CDDP, we overexpressed the high platinum‐resistance genes *COX7B* and *MT1E* in platinum‐resistant cells. Overexpressing these two genes significantly increased the sensitivity to CDDP in 5637PR cells (Figure 2D,E).

**Figure 3:**
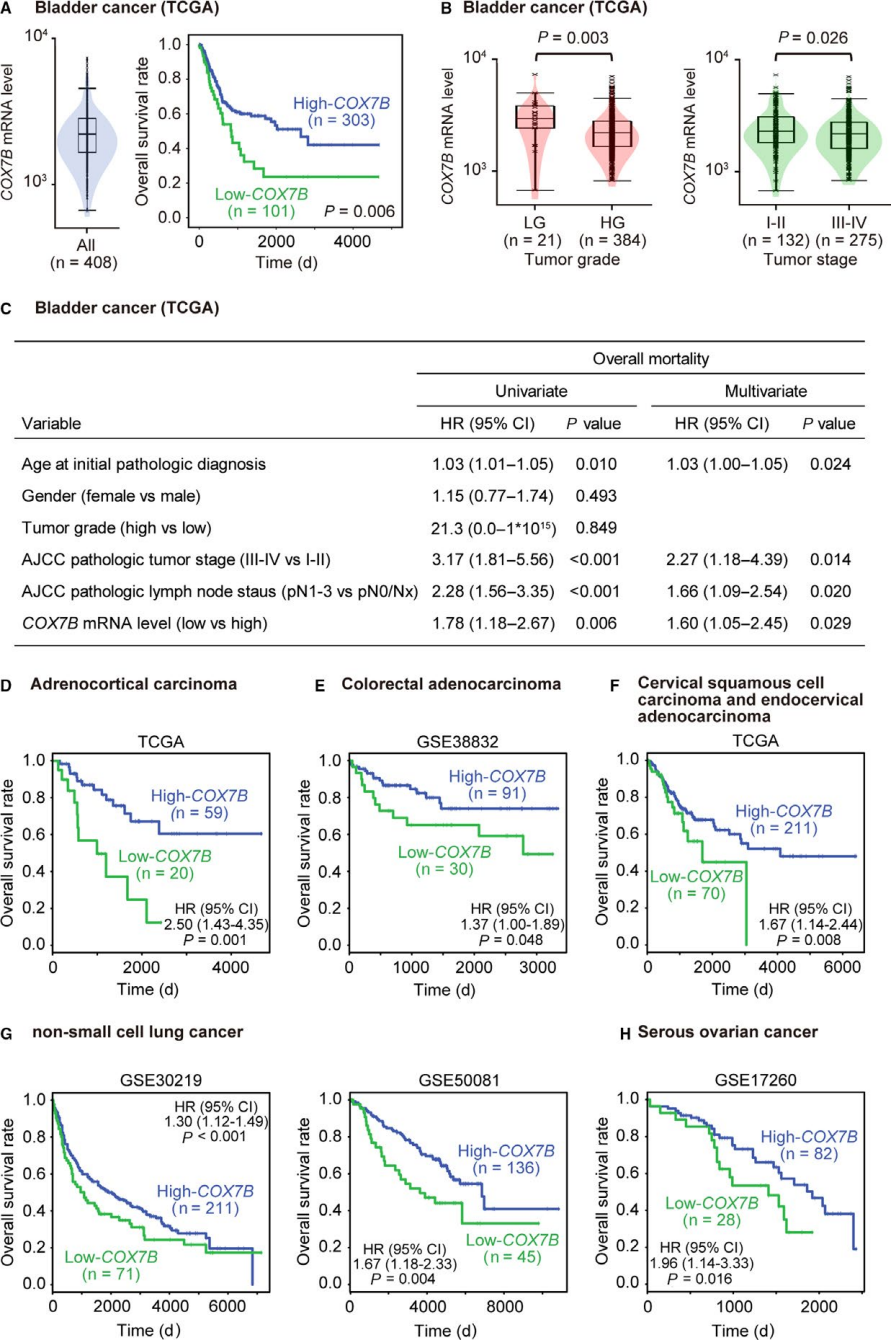
Relationship between high levels of *COX7B* and outcome of multiple cancer types. A, Violin plot with box and dot plots show the heterogeneity of *COX7B* levels in human urinary bladder cancer tissues, obtained from TCGA provisional samples of 408 human urinary bladder cancer patients. Kaplan‐Meier curve shows a significant association between *COX7B* levels and patient survival. Four samples with unknown survivals were excluded from the Kaplan‐Meier analysis. The *P* value from the log‐rank test. B, Relationships between *COX7B* levels and pathological features of tumor grade and stage, obtained from TCGA provisional samples of 408 human urinary bladder cancer patients. Three samples with unknown tumor grade and one sample with unknown tumor stage were excluded. LG, low grade; HG, high grade. The *P* value from the Mann‐Whitney *U* test. C, Predictive risk factors for overall mortality for urinary bladder cancer patients obtained from TCGA provisional samples (n = 408). The multivariate analysis includes the 376 patients (92.2%) that had all required information available. AJCC, American Joint Committee on Cancer; HR, hazard ratio; CI, confidence interval. Kaplan‐Meier analyses of indicated datasets for adrenocortical carcinoma (D), colorectal adenocarcinoma (E), cervical squamous cell carcinoma and endocervical adenocarcinoma (F), non‐small‐cell lung cancer (G), and serous ovarian cancer (H). The *P* values and HR with 95% CI displayed on the survival plots are from the result of proportional hazards analysis using the log‐rank test. Results are obtained using the ProgeneV2 database (http://watson.compbio.iupui.edu/chirayu/proggene).

Then, we sought out to assess the clinical relevance of these gene alterations and determine whether they could stratify patients with cancer. A Kaplan‐Meier analysis of the TCGA provisional dataset for bladder cancer revealed a marked difference in mortality for *COX7B*, showing that low *COX7B* levels significantly predicted poor prognosis (*P = *0.006; Figure 3A). However, the other high platinum‐resistance candidate genes *MT1E* (*P* = 0.299), *LGALS1* (*P* = 0.249), and *KRT17* (*P* = 0.257) showed no significant impact on patient mortality (Figures S6A‐C). Examination of the pathological features of these genes demonstrated that low *COX7B* levels were significantly associated with high tumor grade and advanced stage (Figure 3B). Importantly, multivariate analysis for predicting the overall mortality discovered that a low *COX7B* level (*P* = 0.029) was a risk factor independent of patient age, tumor stage, and lymph node status (Figure 3C). Further, decreased *COX7B* levels significantly predicted patient mortality in the following solid tumors: adrenocortical carcinoma, colorectal adenocarcinoma, cervical squamous cell carcinoma and endocervical adenocarcinoma, non‐small‐cell lung cancer, and ovarian cancer (Figure 3D‐H),^37^, ^38^ which all are plausible candidates for platinum treatment in the clinic. The *COX7B* gene is mapped to chromosome Xq21.1 and encodes a poorly characterized structural subunit of Cytochrome C oxidase (COX), the MRC complex IV (https://ghr.nlm.nih.gov/gene/).

**Figure 4:**
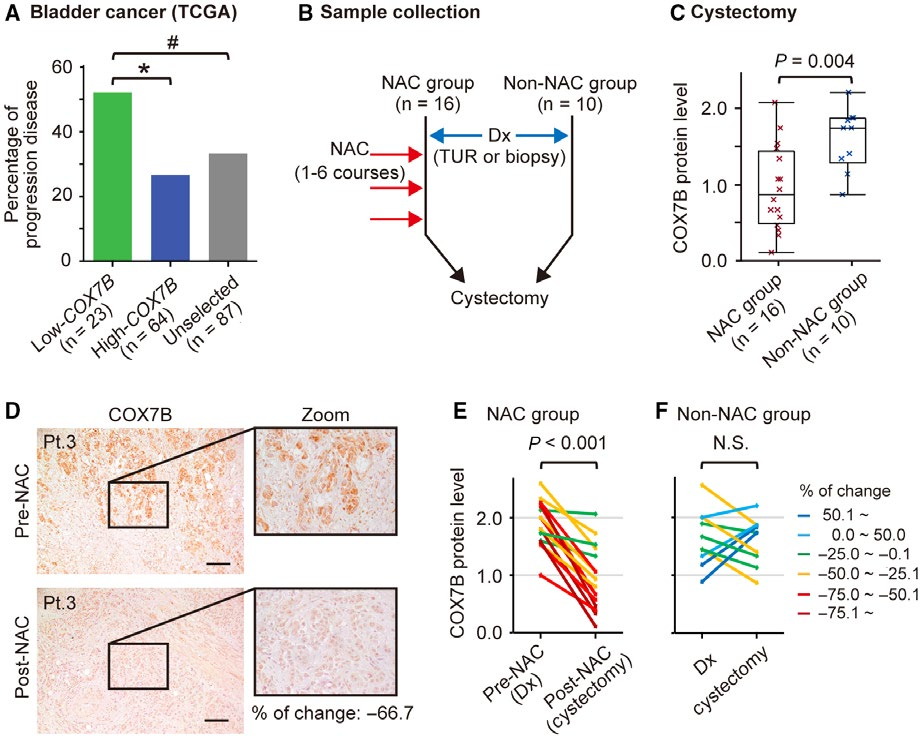
Clinical response and expression profile of high levels of *COX7B* in urinary bladder cancer patients treated with platinum. A, Best responses after platinum chemotherapy in 87 cases from the TCGA cohort of 79 bladder cancer patients plotted in Figure 3A. *P < 0.05, ^#^ *P* < 0.10. The *P* value from the chi‐square test. B, Workflow for obtaining 16 matched clinical urinary bladder cancer sections pre‐ and post‐neoadjuvant CDDP‐based chemotherapy (NAC). Ten human urinary bladder cancer sections without NAC (non‐NAC) were used as controls. Dx, diagnosis. C, Box plots show a significant difference in COX7B protein levels between the NAC (n = 16) and non‐NAC (n = 10) patient groups. The *P* value from the Mann‐Whitney *U* test. D, Representative images of COX7B in matched urinary bladder cancer sections obtained pre‐ and post‐NAC. Scale bars, 100 μm. E,F, Spaghetti plots showing immunolabeling of COX7B in the NAC (n = 16) and non‐NAC (n = 10) patient groups. The *P* value from the paired Student's *t* test. The line within the box is the median. The upper and lower quartiles are bounds of the box, and the minimum and maximum values are the bars

Our next goal was to determine whether CDDP was affecting the COX7B state in intact human tumors. To answer this question, we first analyzed the TCGA dataset for *COX7B* as a biomarker for platinum resistance (Figure 4A). Among the 404 patients with bladder cancer plotted in Figure 3A, 79 (19.6%) were treated with platinum chemotherapy and had clinical data indicating their response to the treatment. Six patients received the treatment twice, and one patient received the treatment three times. Thus, 87 data points were included in the analysis. The result revealed that low *COX7B* levels were significantly associated with poor response to chemotherapy, assuming disease progression under platinum treatment. Then we examined COX7B by immunohistochemistry in a cohort of 16 bladder cancer samples from patients who underwent neoadjuvant CDDP‐based chemotherapy (NAC) before surgery (Figure 4B, Table S6). As a control, we also tested 10 bladder cancer samples from patients without NAC (non‐NAC group, Figure 4B, Table S6). Examination of the surgically treated samples obtained from the NAC and non‐NAC groups showed that the protein levels of COX7B were significantly lower in the NAC group than in the non‐NAC group (*P* = 0.004; Figure 4C). Analyzes of COX7B protein levels in matched pre‐ and posttreatment sections showed a significant difference. In the NAC group, all patients exhibited decreased COX7B protein levels after CDDP compared to before CDDP (*P* < 0.001, Figure 4D,E). The median reduction of COX7B after CDDP was 50.5% whereas no such effect was observed in the non‐NAC group (Figure 4F). Together, these results demonstrated that the COX7B protein level decreases in tumors after treatment with CDDP.

**Figure 5:**
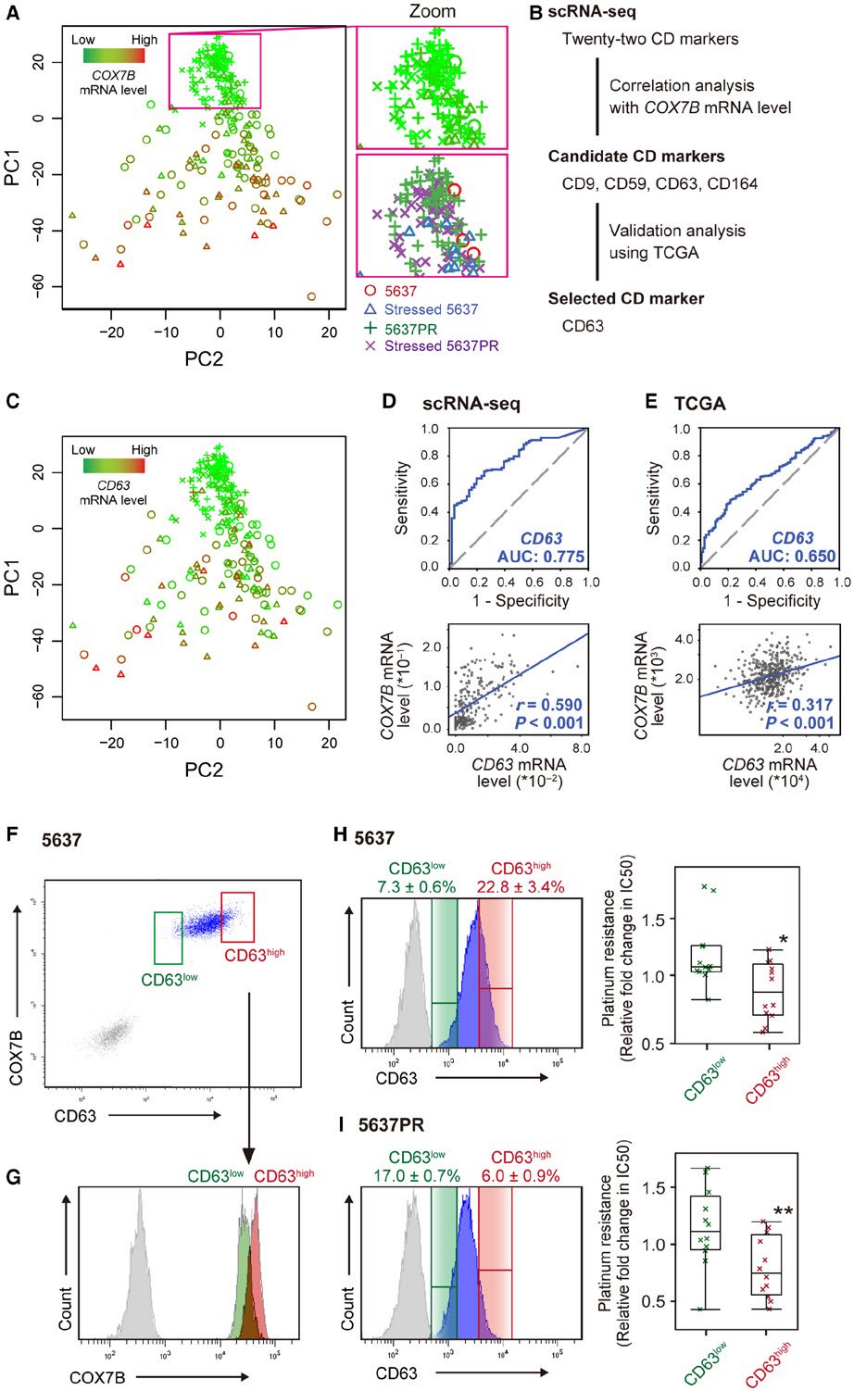
scRNA‐seq identifies innate platinum‐resistant cells in human urinary bladder cancer. A, Principal component analysis (PC1 vs PC2) of the *COX7B* levels listed in Figure 1B (n = 249). The high‐magnification images are of the boxed region with and without pseudo‐coloring. B, Workflow for determining a cell surface marker associated with the *COX7B* level in human urinary bladder cancers. C, Principal component analysis (PC1 vs PC2) of the *CD63* levels listed in Figure 1B (n = 249). Receiver operating characteristic analysis (upper panels) of *CD63* for detecting the low *COX7B* level (<25th percentile) group in all 249 single cells (D) or TCGA provisional for 408 human urinary bladder cancer patients (E). AUC values are indicated. Scatter plots in lower panels show *COX7B* vs *CD63* levels in each dataset. *r*, Spearman's correlation coefficient. F, Scatter plots depicting the FACS analysis of COX7B and CD63 from 5637 cells. Fluorescence‐minus 5637 cells (gray) were used as control. G, The COX7B protein levels of sorted 5637 cells for CD63 were determined by FACS re‐analysis. H, Histogram (blue) plots depicting the FACS analysis of CD63 from 5637 cells. Data are the mean percentages of three independent experiments for sorted 5637 CD63^high^ and CD63^low^ cells. Fluorescence‐minus 5637 cells (gray) are used as a control. Right panel shows effect of CDDP on the viability of FACS‐sorted 5637 CD63^high^ and CD63^low^ cells measured as relative fold change in IC50 (the half maximal inhibitory concentration). Un‐sorted 5637 cells were used as a control. *P < 0.05, compared to sorted 5637 CD63^low^ cells. The *P* value from the two‐tailed Student's *t* test. I, Histogram (blue) depicting the FACS analysis of CD63 from 5637PR cells. Data are the mean percentages of three independent experiments for sorted 5637PR CD63^high^ and CD63^low^ cells. Fluorescence‐minus 5637PR cells (gray) were used as a control. Right panel shows effect of CDDP on the viability of FACS‐sorted 5637PR CD63^high^ and CD63^low^ cells measured as relative fold change in IC50. Un‐sorted 5637PR cells were used as a control. **P < 0.05, compared to sorted 5637PR CD63^low^ cells

The relationship between platinum‐resistance and phenotypical alterations in cancer cells after CDDP treatment remains unclear. This central question assumes the existence of ITH dynamics that give rise to clonal expansion and repopulation during the CDDP treatment.^11^, ^15^, ^22^, ^23^ To address this question, we focused on the ITH profiles^39^ obtained in Figure 1B and stratified the cells according to their levels of *COX7B* (Figure 5A). When analyzing the aggregations resulting from platinum‐resistant 5637PR cells, a small fraction of subclones of parental 5637 cells with low‐*COX7B* were observed (Figure 5A). We hypothesized that these subtypes of parental 5637 cells were cells with acquired platinum‐resistance obtained following CDDP treatment.

To test our hypothesis, we designed a protocol to sort out low‐*COX7B* cells from the other cells using a cell surface marker and subsequently examined their platinum sensitivity (Figure 5B). First, we analyzed the association between *COX7B* and classification determinant (CD) markers in the scRNA‐seq dataset (Figure S7A). Among the 22 CD markers identified, the mRNA levels of four genes significantly correlated with that of *COX7B*, respectively. Second, CD63 was selected after validating the TCGA dataset (Figure S7B,C).^40^, ^41^ Third, the clinical specimens obtained in Figure 4B were examined for CD63. This analysis showed that the patients in the NAC group had significantly lower CD63 protein levels (*P* = 0.010) compared to the corresponding tumor samples before the CDDP treatment (Figure S8A,B).

Analyzing the *CD63* levels for all 249 single cells (Figure 5C) revealed similarities in the pattern of *COX7B* (Figure 5A). To evaluate the diagnostic efficacy of *CD63*, we plotted the results in a receiver operating characteristic graph to detect low‐*COX7B*. Strong tests tend toward the upper left corner; weak tests tend toward the dotted diagonal line. The results yielded an area under the curve (AUC) value of 0.775 from the scRNA‐seq dataset, consistent with the positive correlation between the *COX7B* and *CD63* levels (*r* = 0.590, *P < *0.001; Figure 5D). These results were further confirmed by a TCGA dataset of 408 bladder cancer patients (Figure 5E), producing an AUC value of 0.650 and Spearman's rho of 0.317 (*P < *0.001), respectively.

Using fluorescence‐activated cell sorting (FACS) for CD63, we next sought to isolate CD63^low^ and CD63^high^ cells from bulk parental 5637 cells to further characterize them. The CD63^low^ cells showed weak COX7B protein levels (Figure 5F,G). Investigation of the platinum sensitivity of CD63^low^ cells revealed a significant increase in platinum‐resistance compared to that of CD63^high^ cells when challenging them with CDDP (Figure 5H). This result suggests the existence of a platinum‐resistant subclone in the population of parental platinum‐naïve cells. Further, isolating CD63^high^ cells from 5637PR cells (Figure S9) revealed a significant decrease in platinum‐resistance compared to that of CD63^low^ cells (Figure 5I), yet maintaining the sensitivity to CDDP, even in 5637PR cells. In sum, the results from both cell line experiments demonstrated that COX7B and CD63 were closely related in cancers. Together these genes contribute to the landscape of platinum‐resistance in platinum‐naïve cancers.

## 4 DISCUSSION

Intratumoral heterogeneity profiles are closely associated with acquired drug resistance, leading the natural selection for subclones that will grow and repopulate in cancer cells after receiving chemotherapy.^11^, ^15^, ^22^, ^23^ Platinum‐based chemotherapy has been used for a long time treating patients with solid tumors, and CDDP is the best‐standard agent over the last several decades. Using urinary bladder cancer, this study is the first to show the relationship between platinum‐resistance and the transcriptome dynamics in ITH during treatment with CDDP. These results revealed the *COX7B* gene as a prognostic biomarker for platinum‐resistance in patients with cancer. COX7B has never been linked to platinum‐resistance in urinary bladder cancer, but reports exist in ovarian^42^ and breast^43^ cancer. Knockdown of *COX7B* decreased the sensitivity of CDDP in platinum‐naïve cancer cells, and overexpression of *COX7B* could re‐sensitize the cells to CDDP in platinum‐resistant cancers. The exact molecular mechanisms responsible for rendering cells resistant or sensitive to chemotherapy are unknown. The *COX7B* gene is involved in the metabolism of the cell which indicate that sensitivity or resistance of tumors to neoadjuvant chemotherapy are not only dependent on apoptotic pathways and cell cycle regulation, but that other biologic processes also are required.^43^ Interestingly, *COX7B* is involved in the mitochondrial respiratory chain, which carries out oxidative phosphorylation.^44^ This fact may suggest that a disturbed redox homeostasis is involved in the CDDP resistance of cancer cells. Further, we identified the existence of subclone with low‐*COX7B* that behaved as cancer cells with acquired platinum‐resistance in platinum‐naïve cancers. We speculate that this subclone dominated when the cancer developed its acquired platinum‐resistance. The mechanisms that we here present are novel and highlight the potential advantages of single‐cell analysis in the monitoring platinum‐resistance underlying in cancers treated with CDDP.

Recent developments in the scRNA‐seq have unravel cell‐to‐cell heterogeneity and hidden subclones in the bulk cell population.^24^, ^25^, ^26^, ^27^, ^28^ Applying this sequence technology to cancers has also highlighted the heterogeneous patterns of ITH between different patients and also between tumors coming from the same patient.^2^, ^5^, ^6^, ^8^, ^9^ It is evident that tumors are not uniform; rather, they are entirely heterogeneous at the single‐cell level. This heterogeneity strongly contributes to cell repopulation, which is vital when tumors develop chemoresistance.^23^ However, a vital question that remains to answer is how to use all this knowledge and translate it to patient benefit. In this study, our assessments were further extended to determine an appropriate cell surface and surrogate marker, CD63, to distinguish a platinum‐resistant subclone with low‐*COX7B*. We revealed that assaying CD63 could sort out this subclone from bulk cancer cells, possibly stimulating the development of tailored treatments aiming to determine subclones of cancer cells in the clinic.

scRNA‐seq has also been used to study drug resistance in other cancer types. In lung adenocarcinoma cell lines, ribosomal and housekeeping genes reduce their relative expression diversity during drug treatment with the multi‐tyrosine kinase inhibitor Vandetanib (US trade name Caprelsa).^45^ Interestingly, their result indicates that genes that are directly targeted by the drug of interest, in this case the *EGFR* and *RET* genes, remain constant. In metastatic breast cancer cells subjected to the chemotherapeutic agent Paclitaxel, scRNA‐seq showed that specific transcriptional programs were enacted within untreated, stressed, and drug‐tolerant cell groups, while generating high heterogeneity between single cells within and between groups.^36^ In another study on melanoma cells, the authors observed profound transcriptional variability at the single‐cell level that predicted which cells would ultimately resist drug treatment.^46^ In their study, the reprogramming began with a loss of *SOX10*‐mediated differentiation followed by activation of signaling pathways partially mediated by the transcription factors JUN and/or AP‐1 and TEAD.

In summary, we have applied the scRNA‐seq system to screen urinary bladder cancers and uncovered a dynamic shift in ITH before and after developing platinum‐resistance. This analysis identified a novel platinum‐resistance gene called *COX7B*. Cell population‐based analyses revealed a specific subclone of low‐*COX7B* cells among platinum‐naïve 5637 cells that behaved as cancer cells with acquired platinum‐resistance. We further identified the association between COX7B and CD63 and observed that 59% of the 32 cancer types in TCGA show a positive correlation with *COX7B* (Figure S7C). Although additional studies are required to fully elucidate the functional role of the *COX7B* gene, these scRNA‐seq results could offer a new transcriptome landscape of platinum‐resistance that provides valuable insights into chemosensitivity and cancer stemness at a single‐cell level.^6^, ^11^, ^14^, ^15^, ^22^, ^47^, ^48^, ^49^ Such single‐cell analyses will be instrumental in the design of new clinical diagnostic strategies.

## CONFLICT OF INTEREST

The authors declare no potential conflicts of interest.
