# Supplementary material for: Single‐cell RNA‐seq analysis reveals the platinum resistance gene COX7B and the surrogate marker CD63
Source: Cancer Med. 2018 Oct 26;7(12):6193–204. doi: 10.1002/cam4.1828 (PMC6308066; doi:10.1002/cam4.1828)
Supplement: Supplementary file 1 [file CAM4-7-6193-s001.pdf]

Appendix S1

Small interfering RNA

All sense sequences of the predesigned duplex siRNAs are listed in Table S7. The cells were transiently transfected with 20 nM of the respective siRNAs using Lipofectamine 2000 (Life Technologies). After 12 hours, siRNA was removed by replacing the culture medium with fresh RPMI 1640 containing 10% FBS. Following 48-72 hours, the transfected cells were used for further experimentation. A mock-transfected control was prepared using the transfection reagent only.

Gene overexpression vectors and transfection

Human *COX7B* (456 bp) and *MT1E* (519 bp) cDNA were synthesized with reference to the human *COX7B* and *MT1E* sequence registered in the GenBank database (http://www.ncbi.nlm.gov/genbank), Accession No. NM_001866 for *COX7B* and NM_175617 for *MT1E*, and sub-cloned into the BamHI-XbaI sites of the pBApo-CMV Neo vector (Takara Bio, Otsu, Japan). The *COX7B* and *MT1E* overexpression vectors and the empty control vector were transfected into the cells using TranaIT®-2020 Transfection Reagent (Mirus Bio, Madison, USA) according to the manufacturer's instructions. Following 48-72 hours, the transfected cells were used for further experimentation.

Cell viability assay

All cell lines were seeded at a density of 3-5×10^3 per well onto 96-well culture plates. Following 24 hours of incubation in RPMI 1640 medium supplemented with 10% fetal bovine serum, the cells were incubated for 48 hours with various concentrations of CDDP to investigate platinum sensitivity. At the end of the incubation period, cell viability was determined using the Premix WST-1 Cell Proliferation Assay System (Takara Bio, Otsu, Japan, or Sigma-Aldrich). The absorbance value of each well was determined using a microplate spectrophotometer.

Web-based dataset analysis

We analyzed TCGA urinary bladder cancer samples, for which RNA-seq data are publicly available as provisional (https://www.synapse.org/; access date: November, 2016 and August, 2017). Briefly, the gene-level RNA-seq data (access ID: syn1910448), clinical, pathological, and survival information (access ID: syn2319869 or syn1910531) were obtained for 421 subjects, and matched 408 urinary bladder cancer patients were finally included in the present study. To further study survival outcome associated with the *COX7B* level in other cancer types, ProgeneV2, a web-based genomic/clinical database (www.compbio.iupui.edu/proggene) [1, 2], was used for prognostic assessment (access data: February, 2017). Next, to investigate the cross-cancer relationship between the *COX7B* and *CD63* levels, we analyzed the RNA-seq data from 9721 TCGA samples of 32 cancer types using the Memorial Sloan-Kettering Cancer Center cBioPortal for Cancer Genomics (http://cbioportal.org; access date: October 2016) [3, 4].

Immunohistochemical analysis of clinical samples

Patient selection and preparation

After obtaining institutional review board approval (ethical approval number: 20130095), 52 surgical specimens from 26 patients treated for urinary bladder cancer at Keio University Hospital were

included. In this population, 16 patients were treated with neoadjuvant CDDP-based chemotherapy before cystectomy. All 16 patients were diagnosed as having muscle-invasive urinary bladder cancer based on transurethral resection at diagnosis. Patients received an average of 2.3 cycles of chemotherapy before cystectomy. The surgical specimens were fixed in 10% formalin and embedded in paraffin, and a genitourinary pathologist reassessed all slides. All specimens were histologically confirmed as urothelial carcinoma. Tumors were staged according to the 2002 TMN classification and graded according to the 2004 WHO classification.

Immunohistochemical analysis

Formalin-fixed paraffin-embedded sections (4 µm) were deparaffinized, rehydrated, and washed with distilled water. After antigen retrieval, endogenous peroxidase was quenched, and the sections were blocked with skim milk. Primary antibodies against COX7B (1:100 dilution; # SAB1303153, Sigma-Aldrich) and CD63 (1:100 dilution; # sc-365604, Santa Cruz Biotechnology) were then applied at room temperature for 1 hour. After washing, the sections were incubated with secondary antibodies conjugated to a peroxidase-labeled dextran polymer for 1 hour. Color was developed with 3,3'-diaminobenzamine in 50 mM/L Tris-HCl (pH 5.5) containing 0.005% hydrogen peroxidase. The sections were finally counterstained with hematoxylin. The images were acquired using a fluorescence microscope (IX8, Olympus). The protein levels of COX7B and CD63 were scored as the average of detectable immunoreactions in cancer cells from 0 to 3 (0, no staining; 1, slight staining; 2, medium staining; 3, strong staining) in 3-5 representative areas at 20x magnification. Three investigators blinded to the patient data independently evaluated the immunoreactivity. Finally, the median histoscores for COX7B and CD63 were calculated.

Fluorescence-activated cell sorting (FACS)

The cancer cells were trypsinized into single-cell suspensions. First, to examine the relationship between COX7B and CD63, the cells were fixed, permeabilized, blocked, and subsequently incubated with primary antibodies against COX7B (1:50-100 dilution; # SAB1303153, Sigma-Aldrich) and CD63 (1:100 dilution; # ab59479, Abcam) for 1 hour at room temperature. After washing the unbound antibodies, the cells were suspended in a solution of species-specific secondary antibodies. Second, to examine the cell viability of CDDP, the cells were incubated with Alexa Fluor® 647 anti-CD63 (1:200-300 dilution; # 353016, Biolegend) antibody for 30 min at 4°C, and then used for FACS sorting. Fluorescence-activated cell sorting was performed using a BD FACSAria III Cell Sorter system (Becton Dickinson) with a 488 nm (530/30 filter) and/or 633 nm (660/20 filter) laser. The FACS plots were representative of at least three independent experiments, and fluorescence-minus cells were used as a control.

References

[1] Goswami CP, Nakshatri H. PROGgene: gene expression based survival analysis web application for multiple cancers. Journal of clinical bioinformatics. 2013; 3: 22.

[2] Goswami CP, Nakshatri H. PROGgeneV2: enhancements on the existing database. BMC cancer. 2014; 14: 970.

[3] Cerami E, Gao J, Dogrusoz U, et al. The cBio cancer genomics portal: an open platform for exploring multidimensional cancer genomics data. Cancer discovery. 2012; 2: 401-4.

[4] Gao J, Aksoy BA, Dogrusoz U, et al. Integrative analysis of complex cancer genomics and clinical profiles using the cBioPortal. Science signaling. 2013; 6: pl1.
