# Supplementary material for: Single‐cell RNA‐seq analysis reveals the platinum resistance gene COX7B and the surrogate marker CD63
Source: Cancer Med. 2018 Oct 26;7(12):6193–204. doi: 10.1002/cam4.1828 (PMC6308066; doi:10.1002/cam4.1828)
Supplement: Supplementary file 2 [file CAM4-7-6193-s002.pdf]

Supporting figures

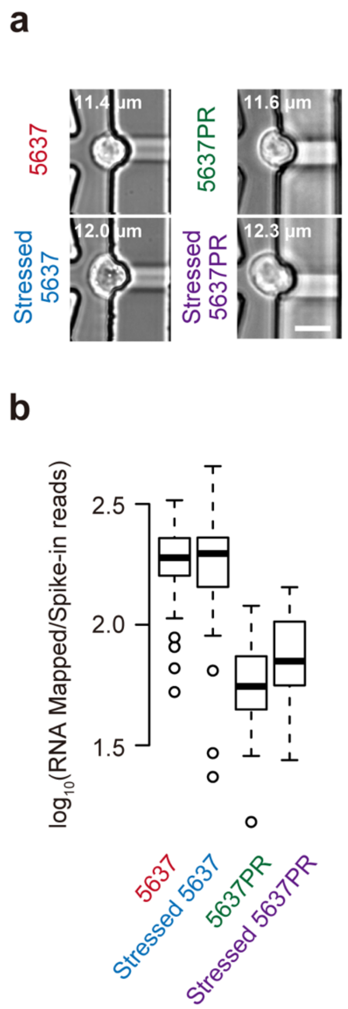

FIGURE S1: Quality control and intra-tumor heterogeneity dynamics assessed by single-cell RNA-seq. (a) Single 5637 and 5637PR cells captured in microfluidic chips. Images were acquired at 20x magnification. Single-cell sizes are indicated. Scale bar, 10 µm. **(b)** Quality measurement of the four libraries of 249 single cells (62 (5637), 63 (stressed 5637), 65 (5637PR), and 59 (stressed 5637PR)) in the Single-cell Tagged Reverse Transcription data for the relative endogenous poly(A)+ transcript amount versus the spike-in amount. The median is shown as the line within the box, the upper and lower quartiles are the bounds of the box, and the minimum and maximum values are the bars.

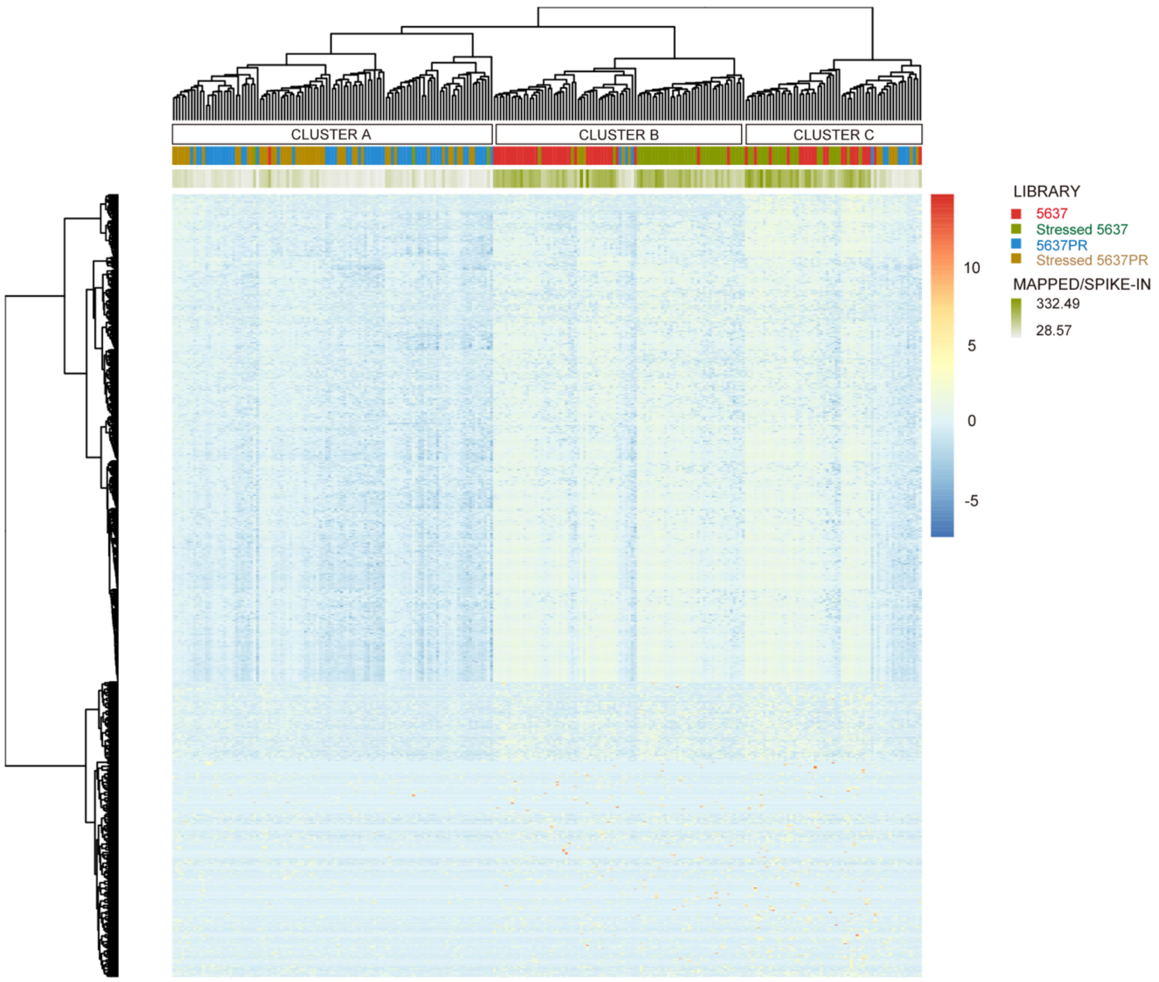

FIGURE S2: Heatmap summarizing intra-tumor heterogeneity dynamics assessed by single-cell RNA-seq. Hierarchical clustering of 1,463 fluctuated genes in all 249 single cells.

a

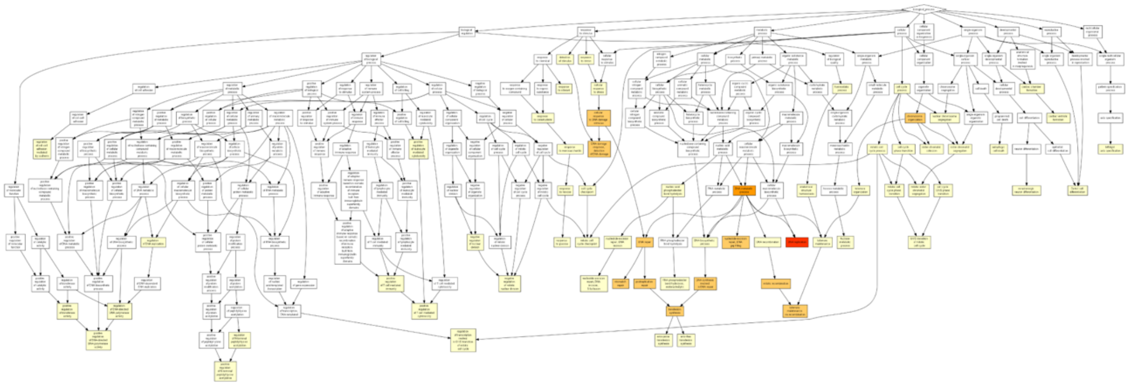

GO Term	Description	*p* value
GO:0006260	DNA replication	8.23E-12
GO:0006259	DNA metabolic process	3.15E-08
GO:0006281	DNA repair	2.08E-07
GO:0006301	postreplication repair	4.11E-07
GO:0006312	mitotic recombination	5.31E-07
GO:0042769	DNA damage response, detection of DNA damage	6.95E-07
GO:0006974	cellular response to DNA damage stimulus	9.11E-07
GO:0051276	chromosome organization	2.26E-06
GO:0000731	DNA synthesis involved in DNA repair	5.48E-06
GO:0019985	translesion synthesis	7.33E-06

b

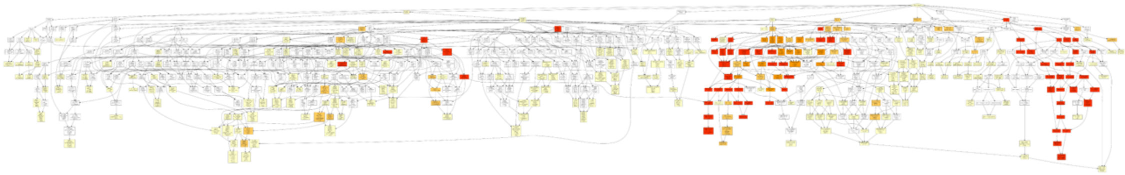

GO Term	Description	*p* value
GO:0006413	translational initiation	8.79E-52
GO:0006614	SRP-dependent cotranslational protein targeting to membrane	9.49E-51
GO:0006613	cotranslational protein targeting to membrane	1.25E-47
GO:0045047	protein targeting to ER	2.19E-47
GO:0072599	establishment of protein localization to endoplasmic reticulum	8.95E-46
GO:0070972	protein localization to endoplasmic reticulum	1.19E-44
GO:0000184	nuclear-transcribed mRNA catabolic process, nonsense-mediated decay	7.35E-43
GO:0019083	viral transcription	2.56E-40
GO:0006612	protein targeting to membrane	2.16E-38
GO:0006412	translation	1.65E-34

FIGURE S3: Up-regulated Gene Ontology term enrichments in differentially expressed genes by CDDP stresses. Directed acyclic graph of up-regulated enriched terms on biological processes and the top five up-regulated enriched terms from 5637 vs. stressed 5637 **(a)** and 5637PR vs. stressed 5637PR **(b)**. GO, gene ontology.

a

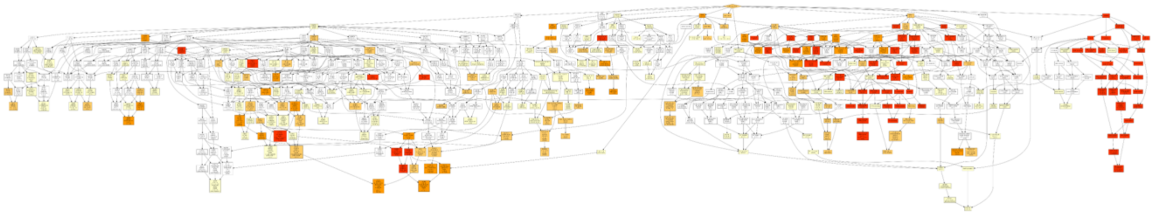

GO Term	Description	*p* value
GO:0006413	translational initiation	8.79E-52
GO:0006614	SRP-dependent cotranslational protein targeting to membrane	9.49E-51
GO:0006613	cotranslational protein targeting to membrane	1.25E-47
GO:0045047	protein targeting to ER	2.19E-47
GO:0072599	establishment of protein localization to endoplasmic reticulum	8.95E-46
GO:0000184	nuclear-transcribed mRNA catabolic process, nonsense-mediated decay	3.33E-46
GO:0019083	viral transcription	3.15E-45
GO:0006413	translational initiation	1.59E-44
GO:0006612	protein targeting to membrane	2.41E-43
GO:0006412	translation	1.97E-40

b

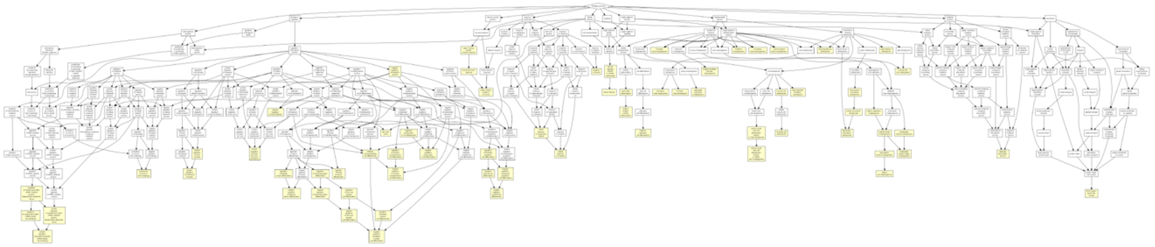

GO Term	Description	*p* value
GO:0008285	negative regulation of cell proliferation	6.40E-05
GO:2000981	negative regulation of inner ear receptor cell differentiation	2.97E-04
GO:2000980	regulation of inner ear receptor cell differentiation	2.97E-04
GO:2000973	regulation of pro-B cell differentiation	2.97E-04
GO:2000974	negative regulation of pro-B cell differentiation	2.97E-04
GO:2000977	regulation of forebrain neuron differentiation	2.97E-04
GO:2000978	negative regulation of forebrain neuron differentiation	2.97E-04
GO:0045977	positive regulation of mitotic cell cycle, embryonic	2.97E-04
GO:0002085	inhibition of neuroepithelial cell differentiation	2.97E-04
GO:0061309	cardiac neural crest cell development involved in outflow tract morphogenesis	2.97E-04

FIGURE S4: Down-regulated Gene Ontology term enrichments in differentially expressed genes by CDDP stresses. Directed acyclic graph of down-regulated enriched terms on biological processes and the top five down-regulated enriched terms from 5637 vs. stressed 5637 **(a)** and 5637PR vs. stressed 5637PR **(b)**. GO, gene ontology.

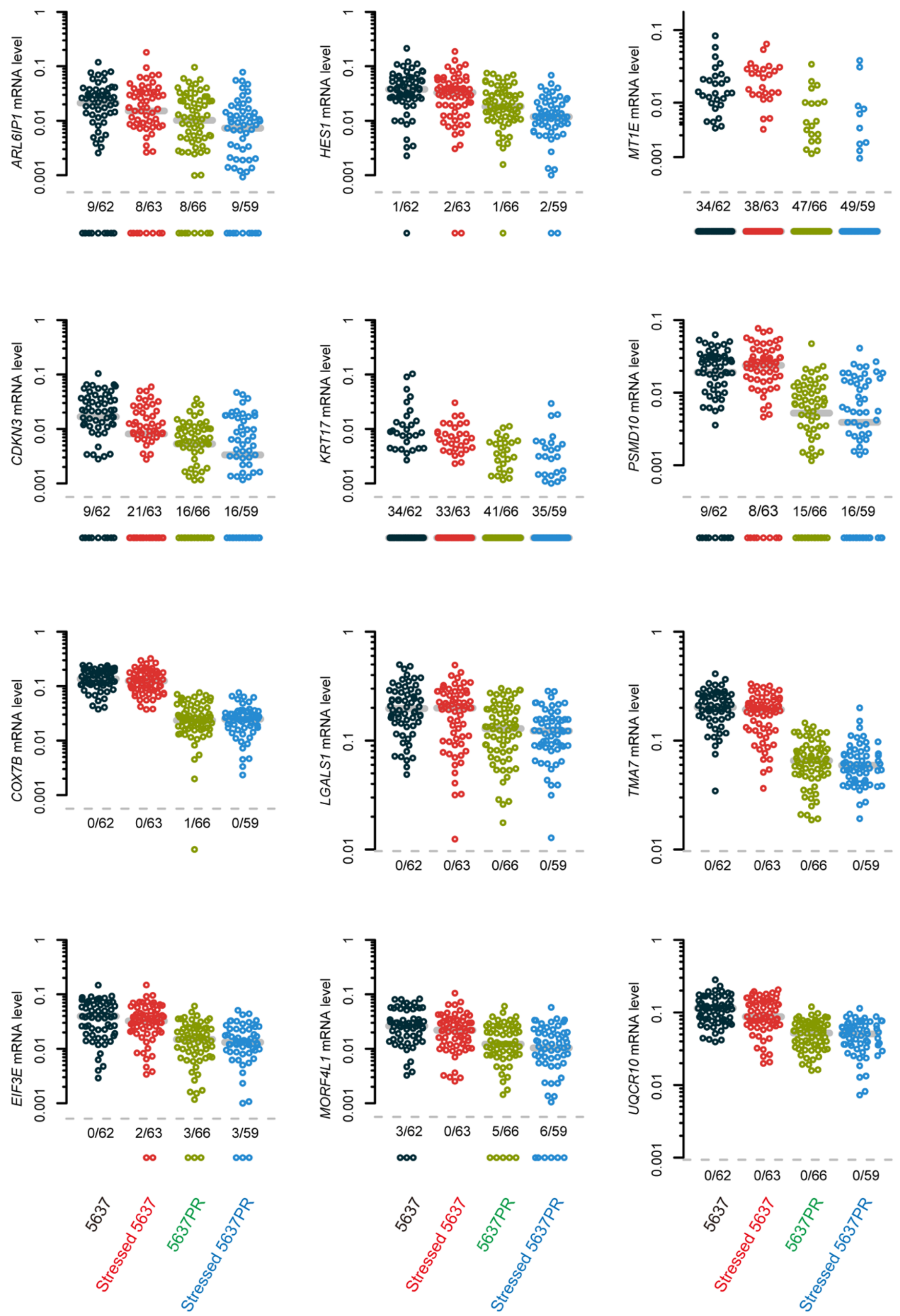

FIGURE S5: Dynamics of differentially expressed genes after acquired platinum-resistance. Dot plots showing the mRNA levels of differently expressed genes listed in Figure 2B (*n* = 12) gradually down-regulated from 5637 to 5637PR cells and 5637PR to stressed 5637PR cells. The lines indicate the median value. The numbers below indicate the zero expression cells in each library.

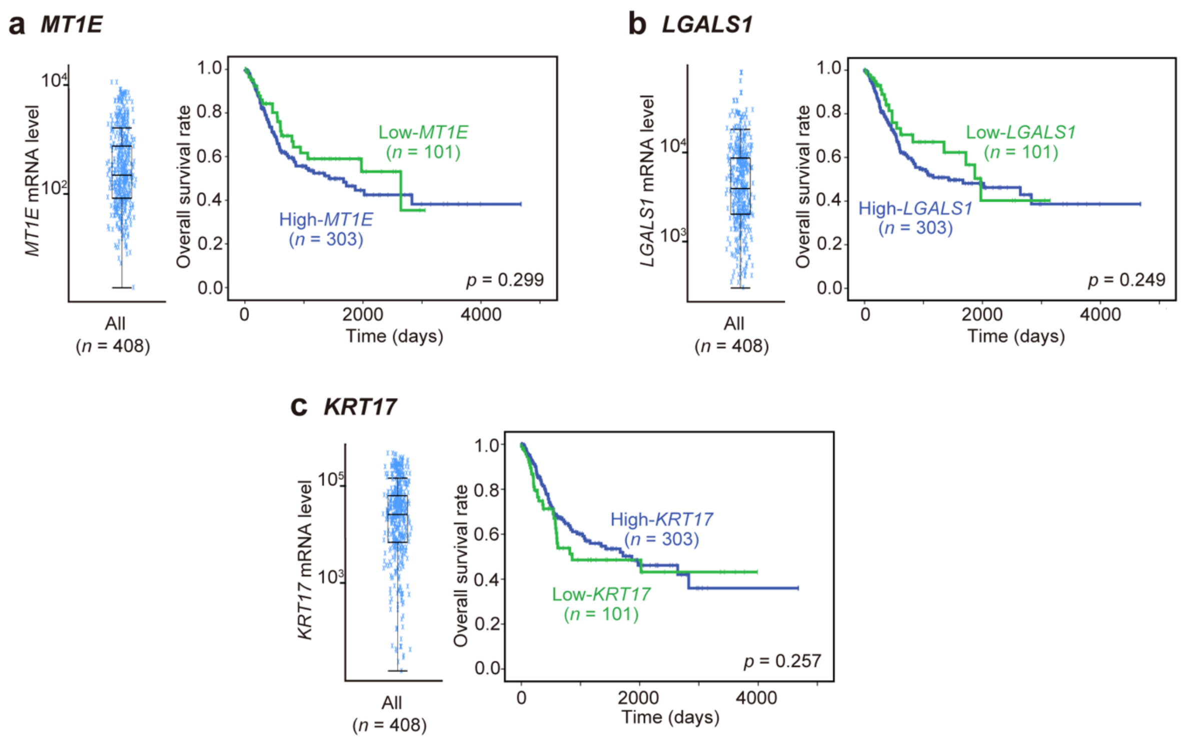

FIGURE S6: Human urinary bladder cancer and outcome of prognosis associated with high platinum-resistance genes. (a-c) Box plots with dot plots show the mRNA level of indicated gene, obtained from TCGA provisional samples of 408 urinary bladder cancer patients. The four samples with unknown survivals were excluded from the Kaplan-Meier analysis. The *p* value from the log-rank test.

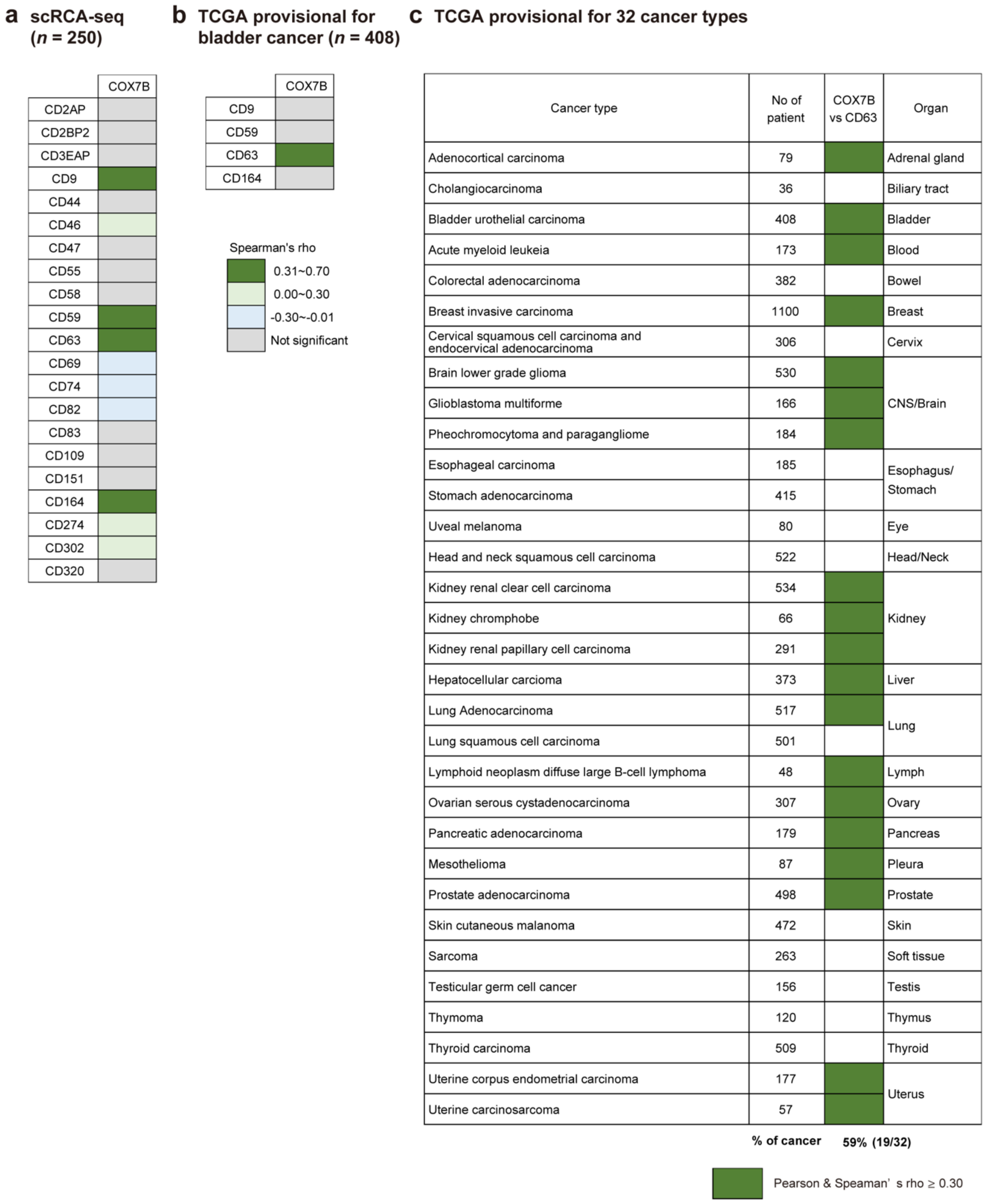

FIGURE S7: Classification determinant (CD) markers associated with high platinum-resistance *COX7B* expression. (a) Relationships between high platinum-resistance *COX7B* and mRNA levels of indicated CD markers in all 249 single cells. **(b)** mRNA profiles between *COX7B* and the four CD markers: *CD9*, *CD59*, *CD63*, and *CD164*, obtained from TCGA provisional samples of 408 urinary bladder cancer patients. **(c)** mRNA profiles of *COX7B* and *CD63*, from TCGA provisional samples obtained from 32 cancer types of cBioPortal for Cancer Genomics. CNS, central nervous system.

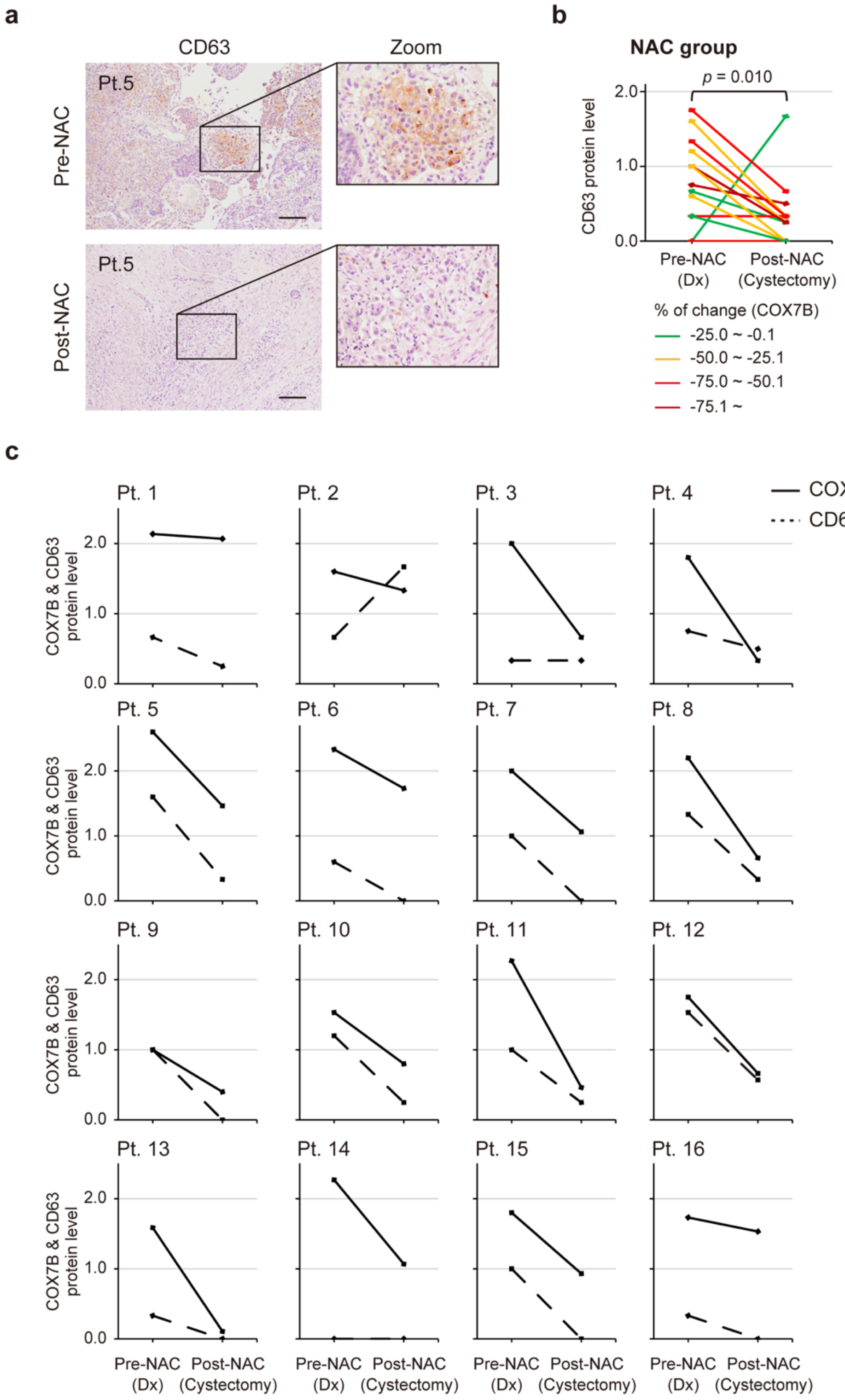

FIGURE S8: Relationship between high platinum-resistance COX7B and CD63 in human urinary bladder cancer. (a) Representative images of CD63 in matched urinary bladder cancer sections obtained pre- and post-neoadjuvant CDDP-based chemotherapy (NAC). **(b)** Spaghetti plots show immunolabeling of CD63 in 16 urinary bladder cancer cohort pre- and post-NAC. Colors indicate % changes in COX7B protein level pre- and post-NAC. The *p* value from the paired Student's *t*-test. Scale bars, 100 µm. **(c)** Individual COX7B and CD63 protein level profiles obtained from 16 bladder cancer cohorts treated with NAC. Dx, diagnosis.

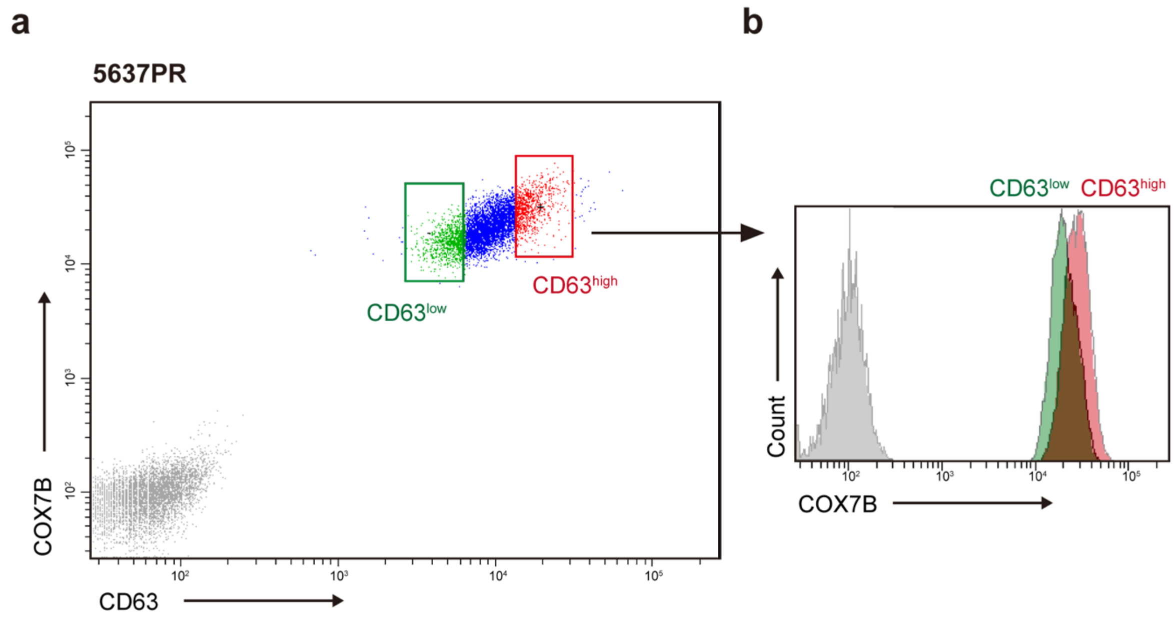

FIGURE S9: Purification of CD63^{low} and CD63^{high} cell population by fluorescence activated cell sorting (FACS). (a) Scatter plot depicting the FACS analysis of COX7B and CD63 from 5637PR cells. Fluorescence-minus 5637PR cells (gray) are used as a control. **(b)** The COX7B protein levels of sorted 5637PR cells for CD63 were determined using FACS re-analysis.
