# Supplementary material for: Single‐cell RNA‐seq analysis reveals the platinum resistance gene COX7B and the surrogate marker CD63
Source: Cancer Med. 2018 Oct 26;7(12):6193–204. doi: 10.1002/cam4.1828 (PMC6308066; doi:10.1002/cam4.1828)
Supplement: Supplementary file 3 [file CAM4-7-6193-s003.pdf]

Supporting tables

TABLE S1: Basic quality information in the RNA sequencing data of 249 single cells.

See below

Table S1. Basic quality information in the RNA sequencing data of 249 single cells

NAME	MAPPED_RATE	SPIKEIN_READS	MAPPED_READS
CDDPMINUS.A01	0.518720847	432	61720
CDDPMINUS.A02	0.526925963	408	106037
CDDPMINUS.A03	0.48737547	528	89526
CDDPMINUS.A04	0.49330486	499	90554
CDDPMINUS.A05	0.531614406	270	88534
CDDPMINUS.A06	0.478348909	441	102525
CDDPMINUS.A07	0.553735873	333	74622
CDDPMINUS.A08	0.500842058	330	56207
CDDPMINUS.A10	0.490849065	411	57984
CDDPMINUS.A11	0.565609727	367	72333
CDDPMINUS.A12	0.604792724	268	50274
CDDPMINUS.B01	0.60856681	249	70029
CDDPMINUS.B03	0.466044672	450	97106
CDDPMINUS.B04	0.544334652	439	99598
CDDPMINUS.B05	0.499095518	438	81115
CDDPMINUS.B07	0.502097902	517	73236
CDDPMINUS.B08	0.503892302	339	71267
CDDPMINUS.B09	0.485206399	388	69549
CDDPMINUS.B10	0.448026466	386	50920
CDDPMINUS.B11	0.518881608	386	89079
CDDPMINUS.C01	0.560561852	350	63015
CDDPMINUS.C02	0.572098621	375	92212
CDDPMINUS.C03	0.488516266	458	91014
CDDPMINUS.C05	0.477316673	452	106886
CDDPMINUS.C07	0.486781221	451	112353
CDDPMINUS.C08	0.487737987	485	95324
CDDPMINUS.C09	0.466187167	428	80242
CDDPMINUS.C10	0.57580015	325	76639
CDDPMINUS.C11	0.587230073	336	77421
CDDPMINUS.C12	0.353764426	441	61399
CDDPMINUS.D01	0.483295316	420	91106
CDDPMINUS.D02	0.434999647	519	67709
CDDPMINUS.D03	0.48149709	496	66267
CDDPMINUS.D04	0.503999179	391	88407
CDDPMINUS.D05	0.472584826	557	81855
CDDPMINUS.D06	0.504035783	366	84177
CDDPMINUS.D07	0.515761539	444	102226
CDDPMINUS.D08	0.457773959	557	88452
CDDPMINUS.D09	0.536664169	400	112451
CDDPMINUS.D11	0.515954036	352	59538
CDDPMINUS.D12	0.54136282	393	68150
CDDPMINUS.E01	0.503593739	453	83518
CDDPMINUS.E05	0.474694429	516	102956
CDDPMINUS.E07	0.531913702	402	85503
CDDPMINUS.E08	0.480158084	455	82616

CDDPMINUS.E10	0.542931676	333	88038
CDDPMINUS.E11	0.548332023	271	48818
CDDPMINUS.E12	0.54271926	297	47908
CDDPMINUS.F02	0.491341943	349	66085
CDDPMINUS.F03	0.508552627	336	78519
CDDPMINUS.F08	0.476019717	496	82568
CDDPMINUS.F09	0.562895377	297	78659
CDDPMINUS.F10	0.498199934	312	64902
CDDPMINUS.F12	0.447733731	217	41123
CDDPMINUS.G01	0.521346117	302	65577
CDDPMINUS.G02	0.488890077	360	73180
CDDPMINUS.G05	0.481008517	359	80871
CDDPMINUS.G06	0.511723644	463	100065
CDDPMINUS.G07	0.524533816	283	75386
CDDPMINUS.G08	0.462557433	322	51645
CDDPMINUS.G12	0.506360339	317	37975
CDDPMINUS.H07	0.429926844	238	30677
CDDPPLUS.A01	0.498975683	354	99131
CDDPPLUS.A04	0.513984232	356	81687
CDDPPLUS.A05	0.505630423	447	98604
CDDPPLUS.A08	0.396066831	354	53290
CDDPPLUS.A09	0.535717036	272	90436
CDDPPLUS.A12	0.531176036	373	69796
CDDPPLUS.B01	0.540583769	319	90917
CDDPPLUS.B02	0.51324573	378	80499
CDDPPLUS.B04	0.477745658	426	38341
CDDPPLUS.B06	0.44007135	475	91530
CDDPPLUS.B07	0.4998552	411	88027
CDDPPLUS.B08	0.540761865	339	81157
CDDPPLUS.B10	0.427497193	398	52928
CDDPPLUS.B12	0.488375848	316	60500
CDDPPLUS.C01	0.52245683	287	61117
CDDPPLUS.C03	0.47476995	344	89619
CDDPPLUS.C06	0.484032549	418	75663
CDDPPLUS.C08	0.472027077	420	88557
CDDPPLUS.C09	0.404429762	479	60476
CDDPPLUS.C11	0.482338165	310	43395
CDDPPLUS.C12	0.529502856	311	65993
CDDPPLUS.D01	0.434503058	425	51224
CDDPPLUS.D02	0.475361521	384	93226
CDDPPLUS.D03	0.473169629	404	113053
CDDPPLUS.D04	0.491243309	343	90488
CDDPPLUS.D05	0.477723142	425	86369
CDDPPLUS.D07	0.441928455	489	80720
CDDPPLUS.D09	0.511509605	427	92106
CDDPPLUS.D10	0.474804403	495	71610
CDDPPLUS.D12	0.520351639	311	75055
CDDPPLUS.E01	0.473471494	345	71578

CDDPPLUS.E05	0.42808776	568	67140
CDDPPLUS.E08	0.421138672	409	59509
CDDPPLUS.E09	0.538868612	313	89914
CDDPPLUS.E11	0.446779746	447	64112
CDDPPLUS.E12	0.504180818	304	68437
CDDPPLUS.F01	0.548774792	286	57578
CDDPPLUS.F03	0.490019608	345	113957
CDDPPLUS.F05	0.445960972	483	72994
CDDPPLUS.F06	0.480958965	392	81751
CDDPPLUS.F07	0.426825526	473	65379
CDDPPLUS.F09	0.534820535	306	89760
CDDPPLUS.F10	0.442498509	457	49724
CDDPPLUS.F11	0.532551967	358	77244
CDDPPLUS.F12	0.488869477	264	52047
CDDPPLUS.G01	0.404560012	418	48760
CDDPPLUS.G02	0.494190298	398	98673
CDDPPLUS.G04	0.458622091	465	86891
CDDPPLUS.G05	0.455944217	455	59503
CDDPPLUS.G06	0.483805188	554	71414
CDDPPLUS.G08	0.489347981	403	86504
CDDPPLUS.G09	0.503416983	339	81767
CDDPPLUS.G11	0.484678319	393	75177
CDDPPLUS.G12	0.489079017	397	50247
CDDPPLUS.H01	0.49479579	362	75633
CDDPPLUS.H03	0.390600481	368	69198
CDDPPLUS.H06	0.411257642	573	70228
CDDPPLUS.H07	0.421933853	330	68444
CDDPPLUS.H08	0.51323041	279	72172
CDDPPLUS.H09	0.500151447	331	87516
CDDPPLUS.H10	0.482035883	365	64534
CDDPPLUS.H11	0.484428975	303	55393
CDDPPLUS.H12	0.528201849	346	59475
CYSMINUS.A01	0.450908985	916	61908
CYSMINUS.A03	0.397921128	1125	49614
CYSMINUS.A04	0.399354953	874	36156
CYSMINUS.A05	0.497061519	1857	82971
CYSMINUS.A06	0.489235462	757	61265
CYSMINUS.A07	0.427329719	1043	50442
CYSMINUS.A10	0.490897468	687	33787
CYSMINUS.A12	0.441627543	805	33864
CYSMINUS.B01	0.408079909	1027	74233
CYSMINUS.B03	0.405024467	1145	40805
CYSMINUS.B04	0.452139303	1118	61344
CYSMINUS.B05	0.473939562	729	51285
CYSMINUS.B06	0.469309485	1048	54943
CYSMINUS.B07	0.443859099	947	67993
CYSMINUS.B09	0.49742396	831	77142
CYSMINUS.B10	0.464425024	725	62389

CYSMINUS.B11	0.557010836	623	50991
CYSMINUS.C01	0.48126763	805	50844
CYSMINUS.C03	0.467545251	835	72196
CYSMINUS.C05	0.418211835	736	33945
CYSMINUS.C06	0.397054289	1068	33482
CYSMINUS.C08	0.431511847	888	56185
CYSMINUS.C09	0.415899552	1114	46671
CYSMINUS.C10	0.45157765	851	84296
CYSMINUS.C12	0.5279607	585	65021
CYSMINUS.D01	0.425187596	939	47030
CYSMINUS.D02	0.467753421	776	42697
CYSMINUS.D04	0.393426052	790	22574
CYSMINUS.D05	0.500093143	840	64429
CYSMINUS.D08	0.428926287	888	27628
CYSMINUS.D09	0.478432872	889	72562
CYSMINUS.D11	0.484721663	691	36136
CYSMINUS.D12	0.414031577	710	37657
CYSMINUS.E02	0.503573054	801	63069
CYSMINUS.E03	0.378574788	971	33809
CYSMINUS.E04	0.441078418	1216	62316
CYSMINUS.E05	0.397103917	934	41053
CYSMINUS.E07	0.427046143	999	63035
CYSMINUS.E08	0.468913565	809	64862
CYSMINUS.E09	0.481873412	981	92166
CYSMINUS.E10	0.45502988	733	39366
CYSMINUS.E11	0.521343915	664	55117
CYSMINUS.F02	0.485185185	1114	35108
CYSMINUS.F03	0.412434997	914	39814
CYSMINUS.F04	0.408931025	1181	49991
CYSMINUS.F05	0.437791713	897	64439
CYSMINUS.F06	0.433856583	1176	60418
CYSMINUS.F12	0.428124417	841	41320
CYSMINUS.G01	0.525291535	638	76442
CYSMINUS.G04	0.450540119	931	55179
CYSMINUS.G05	0.433410347	1112	63593
CYSMINUS.G06	0.435843232	962	49276
CYSMINUS.G07	0.42418795	1156	92028
CYSMINUS.G09	0.43978443	1139	74178
CYSMINUS.G12	0.502202796	762	57908
CYSMINUS.H01	0.488101411	861	54600
CYSMINUS.H02	0.521481428	739	43199
CYSMINUS.H03	0.416267505	1127	50057
CYSMINUS.H04	0.518959689	725	55113
CYSMINUS.H06	0.476473408	923	60626
CYSMINUS.H08	0.392940146	856	58330
CYSMINUS.H09	0.381329281	1121	39651
CYSMINUS.H10	0.517939382	951	71977
CYSMINUS.H11	0.518561309	1760	78547

CYSMINUS.H12	0.47702257	842	40348
CYSPLUS.A03	0.445740743	1175	82602
CYSPLUS.A04	0.485830182	1155	61921
CYSPLUS.A05	0.424693868	1326	74775
CYSPLUS.A06	0.420810815	1105	60266
CYSPLUS.A08	0.429280117	1139	53305
CYSPLUS.A09	0.512931004	880	72372
CYSPLUS.A11	0.468531352	1041	56183
CYSPLUS.A12	0.462336279	986	44265
CYSPLUS.B01	0.47793744	1021	47030
CYSPLUS.B02	0.459824729	949	46646
CYSPLUS.B03	0.448911166	1149	79798
CYSPLUS.B05	0.406955855	1145	54141
CYSPLUS.B07	0.545855451	884	94195
CYSPLUS.B09	0.513274551	829	84756
CYSPLUS.B10	0.505201954	708	73421
CYSPLUS.B12	0.48910842	868	72929
CYSPLUS.C02	0.509371644	1058	74952
CYSPLUS.C03	0.500229996	938	93523
CYSPLUS.C04	0.515589163	980	102958
CYSPLUS.C06	0.475634384	850	105041
CYSPLUS.C07	0.433244565	1372	88339
CYSPLUS.C08	0.396332151	1270	65914
CYSPLUS.C09	0.445096829	1143	70652
CYSPLUS.C10	0.493700614	795	78412
CYSPLUS.C12	0.486502179	911	62841
CYSPLUS.D01	0.546462145	638	79231
CYSPLUS.D02	0.403137626	1293	62161
CYSPLUS.D03	0.483566829	1007	114733
CYSPLUS.D05	0.490654543	834	61191
CYSPLUS.D07	0.459685076	868	53745
CYSPLUS.D09	0.44084611	1152	59147
CYSPLUS.D11	0.453938408	997	58327
CYSPLUS.E02	0.44291031	1204	74261
CYSPLUS.E03	0.471367172	1107	101557
CYSPLUS.E05	0.521931431	815	92635
CYSPLUS.E07	0.518095906	929	94624
CYSPLUS.E08	0.45032433	1076	60121
CYSPLUS.E09	0.395621905	772	85412
CYSPLUS.E10	0.427107014	742	50915
CYSPLUS.E12	0.476880164	627	71976
CYSPLUS.F01	0.425569166	1188	66845
CYSPLUS.F04	0.472852509	901	61372
CYSPLUS.F05	0.437402095	1197	96333
CYSPLUS.F07	0.451708558	1242	80279
CYSPLUS.F09	0.447841854	1119	69776
CYSPLUS.F10	0.48828541	1008	107289
CYSPLUS.F12	0.480428987	872	72257

CYSPLUS.G02	0.526055145	784	84768
CYSPLUS.G03	0.443833606	782	44161
CYSPLUS.G06	0.488380915	1016	110714
CYSPLUS.G10	0.480300339	865	59490
CYSPLUS.G12	0.439214405	1039	57787
CYSPLUS.H01	0.555532133	624	71152
CYSPLUS.H03	0.446761371	1182	58276
CYSPLUS.H04	0.496632292	1282	99689
CYSPLUS.H05	0.531137554	936	91370
CYSPLUS.H10	0.512946012	1197	82374
CYSPLUS.H11	0.504235522	982	78632
CYSPLUS.H12	0.568898288	854	72365

MAPPED/SPIKEIN	Estimated total transcript counts
142.8703704	996520.8335
259.8946078	1812764.889
169.5568182	1182658.807
181.4709419	1265759.82
327.9037037	2287128.333
232.4829932	1621568.878
224.0900901	1563028.378
170.3242424	1188011.591
141.080292	984035.0367
197.0926431	1374721.186
187.5895522	1308437.127
281.2409639	1961655.723
215.7911111	1505143
226.8747153	1582451.139
185.1940639	1291728.596
141.655706	988048.5494
210.2271386	1466334.292
179.25	1250268.75
131.9170984	920121.7613
230.7746114	1609652.915
180.0428571	1255798.928
245.8986667	1715143.2
198.720524	1386075.655
236.4734513	1649402.323
249.1197339	1737610.144
196.5443299	1370896.701
187.4813084	1307682.126
235.8123077	1644790.846
230.4196429	1607177.009
139.2267574	971106.6329
216.9190476	1513010.357
130.460501	909961.9945
133.6028226	931879.6876
226.1048593	1577081.394
146.956912	1025024.461
229.9918033	1604192.828
230.2387387	1605915.202
158.8007181	1107635.009
281.1275	1960864.313
169.1420455	1179765.767
173.4096692	1209532.443
184.3664459	1285955.96
199.5271318	1391701.744
212.6940299	1483540.859
181.5736264	1266476.044

264.3783784	1844039.189
180.1402214	1256478.044
161.3063973	1125112.121
189.3553009	1320753.224
233.6875	1629970.313
166.4677419	1161112.5
264.8451178	1847294.697
208.0192308	1450934.135
189.5069124	1321810.714
217.1423841	1514568.129
203.2777778	1417862.5
225.2674095	1571240.181
216.1231102	1507458.694
266.3816254	1858011.837
160.3881988	1118707.687
119.7949527	835569.7951
128.894958	899042.3321
280.0310734	1953216.737
229.4578652	1600468.61
220.590604	1538619.463
150.5367232	1049993.644
332.4852941	2319084.926
187.1206434	1305166.488
285.0062696	1987918.73
212.9603175	1485398.215
90.00234742	627766.3733
192.6947368	1344045.789
214.1776156	1493888.869
239.4011799	1669823.23
132.9849246	927569.8491
191.4556962	1335403.481
212.9512195	1485334.756
260.5203488	1817129.433
181.0119617	1262558.433
210.85	1470678.75
126.2546973	880626.5137
139.983871	976387.5002
212.1961415	1480068.087
120.5270588	840676.2351
242.7760417	1693362.891
279.8341584	1951843.255
263.8134111	1840098.542
203.2211765	1417467.706
165.0715746	1151374.233
215.704918	1504541.803
144.6666667	1009050
241.3344051	1683307.476
207.4724638	1447120.435

118.2042254	824474.4722
145.4987775	1014853.973
287.2651757	2003674.601
143.4272931	1000405.369
225.1217105	1570223.931
201.3216783	1404218.706
330.3101449	2303913.261
151.126294	1054105.901
208.5484694	1454625.574
138.2219873	964098.3614
293.3333333	2046000
108.8052516	758916.6299
215.7653631	1504963.408
197.1477273	1375105.398
116.6507177	813638.756
247.9221106	1729256.721
186.8623656	1303365
130.7758242	912161.3738
128.9061372	899120.307
214.6501241	1497184.616
241.20059	1682374.115
191.2900763	1334248.282
126.5667506	882803.0854
208.9309392	1457293.301
188.0380435	1311565.353
122.5619546	854869.6333
207.4060606	1446657.273
258.6810036	1804300
264.3987915	1844181.571
176.8054795	1233218.22
182.8151815	1275135.891
171.8930636	1198954.119
67.58515284	471406.4411
44.10133333	307606.8
41.36842105	288544.7368
44.68012924	311643.9014
80.93130779	564495.8718
48.36241611	337327.8524
49.18049491	343033.952
42.06708075	293417.8882
72.28140214	504162.7799
35.63755459	248571.9433
54.86940966	382714.1324
70.34979424	490689.8148
52.42652672	365675.0239
71.79831045	500793.2154
92.83032491	647491.5162
86.0537931	600225.2069

81.84751204	570886.3965
63.16024845	440542.7329
86.46227545	603074.3713
46.12092391	321693.4443
31.35018727	218667.5562
63.2713964	441317.9899
41.89497307	292217.4372
99.05522914	690910.2233
111.1470085	775250.3843
50.08519702	349344.2492
55.02190722	383777.8029
28.57468354	199308.4177
76.70119048	534990.8036
31.11261261	217010.473
81.62204724	569313.7795
52.29522431	364759.1896
53.03802817	369940.2465
78.73782772	549196.3483
34.81874356	242860.7363
51.24671053	357445.8059
43.95396146	306578.8812
63.0980981	440109.2342
80.17552534	559224.2892
93.95107034	655308.7156
53.7053206	374594.6112
83.00753012	578977.5226
31.51526032	219818.9407
43.56017505	303832.221
42.32938188	295247.4386
71.83835006	501072.4917
51.37585034	358346.5561
49.13198573	342695.6005
119.815047	835709.9528
59.26852846	413397.986
57.18794964	398885.9487
51.22245322	357276.6112
79.60899654	555272.7509
65.12554873	454250.7024
75.99475066	530063.3859
63.41463415	442317.0732
58.45602165	407730.751
44.41614907	309802.6398
76.01793103	530225.0689
65.6836403	458143.3911
68.14252336	475294.1004
35.37109723	246713.4032
75.68559411	527907.0189
44.62897727	311287.1165

47.9192399	334236.6983
70.29957447	490339.5319
53.61125541	373938.5065
56.39140271	393330.0339
54.53936652	380412.0815
46.79982441	326428.7753
82.24090909	573630.3409
53.97022094	376442.2911
44.89350913	313132.2262
46.06268364	321287.2184
49.15279241	342840.7271
69.44995648	484413.4464
47.28471616	329810.8952
106.5554299	743224.1236
102.238842	713115.923
103.7019774	723321.2924
84.01958525	586036.6071
70.84310019	494130.6238
99.70469083	695440.2185
105.0591837	732787.8063
123.5776471	861954.0885
64.38702624	449099.508
51.9007874	362007.9921
61.8127734	431144.0945
98.63144654	687954.3396
68.98024149	481137.1844
124.1865204	866200.9798
48.07501933	335323.2598
113.9354518	794699.7763
73.3705036	511759.2626
61.91820276	431879.4643
51.34288194	358116.6015
58.50250752	408054.99
61.67857143	430208.0357
91.74074074	639891.6667
113.6625767	792796.4725
101.8557589	710443.9183
55.87453532	389724.8839
110.6373057	771695.2073
68.61859838	478614.7237
114.7942584	800689.9523
56.26683502	392461.1743
68.1154273	475105.1054
80.47869674	561338.9098
64.63687601	450842.2102
62.35567471	434930.8311
106.4375	742401.5625
82.86353211	577973.1365

108.122449	754154.0818
56.47186701	393891.2724
108.9704724	760069.045
68.77456647	479702.6011
55.61790183	387934.8653
114.025641	795328.846
49.30287648	343887.5634
77.76053042	542379.6997
97.61752137	680882.2116
68.81704261	479998.8722
80.07331976	558511.4053
84.73653396	591037.3244

TABLE S2: A total of 1,132 differentially expressed (DE) genes between cell populations of 5637 vs. 5637PR.

See below

Table S2. A total of 1,132 differentially expressed (DE) genes between cell populations of 5637 vs. 5637PR.

Gene symbol	DE score	*p* value	*q* value
AATF	-253.3	0.178690503	0.037729366
ABRACL	-1683.7	0.000982808	0
ACAD8	12.3	0.960897461	0
ACAT1	-659.15	0.001426724	0
ACAT2	-927.8	0.000982808	0
ACP1	-1372.5	0.000982808	0
ACTB	-1587.1	0.000982808	0
ACTG1	-1350.2	0.000982808	0
ACTL6A	-1102.05	0.000982808	0
ACTR1A	-497.6	0.010435484	0.000750079
ADH5	-850.5	0.000982808	0
ADIRF	-1683.35	0.000982808	0
ADSL	-516	0.008138437	0.000404178
AGK	-324.25	0.087245571	0.014214327
AHSA1	-713.15	0.001077855	0
AIMP2	-1076.9	0.000982808	0
AK2	-995.5	0.000982808	0
AKR1B1	-1492.7	0.000982808	0
ALDOA	-1339.3	0.000982808	0
ALG14	-244.55	0.193995329	0.041820213
ALG6	81.5	0.693474105	0
AMD1	-1377.95	0.000982808	0
AMZ2	-453.65	0.018825878	0.001739016
ANAPC11	-1689.4	0.000982808	0
ANAPC13	-1102.2	0.000982808	0
ANAPC15	-739.4	0.001001799	0
ANAPC5	-675.1	0.001278047	0
ANP32B	-1126.25	0.000982808	0
ANP32E	-342.25	0.073009557	0.011273954
ANXA1	-1969.7	0.000982808	0
ANXA2	-1955.2	0.000982808	0
ANXA3	-1300.2	0.000982808	0
ANXA5	-1552.8	0.000982808	0
AP2S1	-541.45	0.005809772	0.000404178
APEX1	-1491.4	0.000982808	0
APOO	-963.45	0.000982808	0
APRT	-554.05	0.004882138	0.000404178
ARFRP1	5.75	0.981676648	0
ARHGDIB	-1214.2	0.000982808	0
ARL3	-497.55	0.010437044	0.000750079
ARL4D	-350.4	0.066941065	0.010057307
ARL6IP1	-441.9	0.021968421	0.0023441
ARMCX5	19.2	0.93619718	0
ARMT1	-677.3	0.001261924	0
ARPC1A	-1406.85	0.000982808	0

ARPC2	-867.2	0.000982808	0
ARPC3	-1663.55	0.000982808	0
ASB9	-454.2	0.018695295	0.001739016
ATAD2	-865.45	0.000982808	0
ATG3	-694.35	0.001168908	0
ATIC	-692.6	0.001173478	0
ATOX1	-1263.05	0.000982808	0
ATP5A1	-1614	0.000982808	0
ATP5B	-1805.15	0.000982808	0
ATP5C1	-1409.75	0.000982808	0
ATP5E	-1905.45	0.000982808	0
ATP5F1	-1663.85	0.000982808	0
ATP5G1	-1685.6	0.000982808	0
ATP5G3	-1807.05	0.000982808	0
ATP5H	-1756.75	0.000982808	0
ATP5I	-1722.55	0.000982808	0
ATP5J	-1863	0.000982808	0
ATP5L	-1901.55	0.000982808	0
ATP5O	-1730.85	0.000982808	0
ATP6V0B	-1264.5	0.000982808	0
ATP6V0E1	-1772.7	0.000982808	0
ATP6V1D	-1291.75	0.000982808	0
ATP6V1E1	-240.4	0.201892738	0.044148535
ATP6V1F	-1572.9	0.000982808	0
ATP6V1G1	-1663.95	0.000982808	0
ATPIF1	-918.65	0.000982808	0
AURKA	-728.5	0.001015943	0
B2M	-1647.2	0.000982808	0
BAG5	-482.3	0.012831261	0.001091209
BANF1	-1709.1	0.000982808	0
BCAP29	-374.3	0.050611715	0.006870833
BCAP31	-1621.7	0.000982808	0
BCAS2	-1215.85	0.000982808	0
BCLAF1	-432.25	0.025010417	0.002655394
BCS1L	-268	0.155533584	0.03141909
BET1	-1206.55	0.000982808	0
BEX3	-1834.65	0.000982808	0
BFAR	-255.05	0.175868199	0.036973682
BIRC3	332.5	0.080622727	0
BNIP1	-256.5	0.173803159	0.036253082
BOLA3	-1102.85	0.000982808	0
BRIX1	-959.6	0.000982808	0
BRK1	-1731.2	0.000982808	0
BSG	-882.2	0.000982808	0
BTBD10	-444.05	0.021370752	0.002035205
BTF3	-1804.35	0.000982808	0
BTF3L4	-734.2	0.001008961	0
BUB1	-270.95	0.150835714	0.030099681
BUB3	-612.25	0.002336357	0

BUD31	-1728.85	0.000982808	0
BZW1	-1278.55	0.000982808	0
BZW2	-1298.9	0.000982808	0
C11orf58	-1745.8	0.000982808	0
C11orf73	-1196.85	0.000982808	0
C11orf74	-496.3	0.010599031	0.000750079
C11orf98	-1232.15	0.000982808	0
C12orf57	-1138.55	0.000982808	0
C14orf1	-985.15	0.000982808	0
C14orf166	-1770.9	0.000982808	0
C14orf2	-1866	0.000982808	0
C15orf48	-787	0.000982808	0
C19orf33	-1441.65	0.000982808	0
C19orf48	-1136.65	0.000982808	0
C19orf53	-1617.2	0.000982808	0
C1QBP	-267.95	0.15556793	0.03141909
C1orf174	-352.7	0.065095613	0.009812006
C1orf43	-649.15	0.001571097	0
C3orf14	-582.55	0.003358038	0
C6orf62	-247.15	0.189330263	0.040526037
C7orf73	-1290.85	0.000982808	0
C8orf59	-1778.45	0.000982808	0
C8orf76	-587.75	0.00311775	0
C9orf78	-1190.95	0.000982808	0
CA2	-559.25	0.004575319	0
CACYBP	-1609.45	0.000982808	0
CALM1	-924.2	0.000982808	0
CALM2	-1722.2	0.000982808	0
CALU	-1784	0.000982808	0
CAPZA2	-1394.6	0.000982808	0
CARD19	4	0.987034809	0
CAV1	-1952.35	0.000982808	0
CAV2	-1267.05	0.000982808	0
CBX1	-686.15	0.001201646	0
CBX3	-1926.55	0.000982808	0
CBX5	-919.95	0.000982808	0
CCL20	28.45	0.90583472	0
CCNB1	-967.45	0.000982808	0
CCNB1IP1	-348.3	0.068443053	0.010057307
CCNB2	-614.85	0.002262044	0
CCNE2	-1059.3	0.000982808	0
CCNG1	-1241.9	0.000982808	0
CCNI	-277.4	0.141746476	0.027417891
CCT2	-1790.4	0.000982808	0
CCT3	-1380.1	0.000982808	0
CCT4	-715.5	0.00107367	0
CCT5	-1383.75	0.000982808	0
CCT6A	-1408.6	0.000982808	0

CCT7	-1240.6	0.000982808	0
CCT8	-1718.35	0.000982808	0
CD164	-916.25	0.000982808	0
CD59	-1529	0.000982808	0
CD63	-1704.95	0.000982808	0
CD9	-1135.4	0.000982808	0
CDC123	-1311.1	0.000982808	0
CDC23	-248.05	0.187741711	0.039916603
CDC26	-1773.1	0.000982808	0
CDC5L	-664.3	0.001376089	0
CDC6	-997.85	0.000982808	0
CDCP1	31.7	0.893624482	0
CDIPT	10.45	0.966719143	0
CDK1	-712.8	0.001077855	0
CDK2	3.05	0.989504059	0
CDK7	-340.35	0.074317824	0.01148073
CDKN3	-1005.05	0.000982808	0
CENPF	-533.85	0.006431101	0.000404178
CENPP	25.5	0.917677375	0
CENPW	-1324.8	0.000982808	0
CEP41	-744.15	0.000999091	0
CFL1	-1059.95	0.000982808	0
CFL2	-524.3	0.007310914	0.000404178
CHCHD2	-1468.5	0.000982808	0
CHCHD3	-1149.6	0.000982808	0
CHMP1B	-675.5	0.001275444	0
CHORDC1	-1424.45	0.000982808	0
CIB1	-453.65	0.018825878	0.001739016
CKAP2	-553.3	0.004922414	0.000404178
CKS1B	-1180.3	0.000982808	0
CKS2	-1380.5	0.000982808	0
CLDN7	-944.45	0.000982808	0
CLIC1	-1684.9	0.000982808	0
CLINT1	-287.55	0.127670156	0.02389855
CLK1	-251.75	0.181374715	0.038536984
CLK2	12.7	0.959800409	0
CLNS1A	-844.5	0.000982808	0
CLSPN	-1168.6	0.000982808	0
CLTA	-712.25	0.001079447	0
CMC2	-768.1	0.00098324	0
CMSS1	-1505.6	0.000982808	0
CNBP	-1715.95	0.000982808	0
CNIH1	-1254.25	0.000982808	0
CNIH4	-1564.9	0.000982808	0
CNN3	-744.95	0.000999091	0
CNPY2	-1100	0.000982808	0
COA1	-1323.9	0.000982808	0
COA3	-1200.15	0.000982808	0

COMMD6	-1761.75	0.000982808	0
COPB2	-1065	0.000982808	0
COPS3	-521.35	0.00757327	0.000404178
COPS4	-611.95	0.002338289	0
COPS6	-1178.95	0.000982808	0
COPS7A	-283.75	0.132734121	0.024978393
COPS8	-1148.05	0.000982808	0
COX14	-1314.15	0.000982808	0
COX16	-1803.85	0.000982808	0
COX17	-1614.4	0.000982808	0
COX4I1	-1741.9	0.000982808	0
COX5A	-551.95	0.005012897	0.000404178
COX5B	-1069.1	0.000982808	0
COX6A1	-1768.55	0.000982808	0
COX6B1	-1903.65	0.000982808	0
COX6C	-1848.9	0.000982808	0
COX7A2L	-1204.45	0.000982808	0
COX7B	-1961.35	0.000982808	0
COX7C	-1918.4	0.000982808	0
COX8A	-1711.75	0.000982808	0
CPSF6	-337.8	0.076349747	0.011963048
CSDE1	-1304.2	0.000982808	0
CSF2	-304.85	0.107328291	0.01885745
CSNK2A1	-453.1	0.018958428	0.001739016
CSPP1	35	0.880350622	0
CSRP2	-1230.25	0.000982808	0
CSTB	-1182.1	0.000982808	0
CTNNAL1	-478.5	0.013514437	0.001091209
CTNNBL1	-686.05	0.001201646	0
CTPS1	-670	0.001316147	0
CTSC	-1426.75	0.000982808	0
CTSL	-814.25	0.000982808	0
CUTA	-1205.75	0.000982808	0
CWC15	-1414.45	0.000982808	0
CYB5A	-443.95	0.02138453	0.002035205
CYB5B	-590.05	0.003046634	0
CYCS	-1993.2	0.000982808	0
CYLD	36.25	0.877033458	0
DAD1	-1642.45	0.000982808	0
DBF4	-955.5	0.000982808	0
DBI	-1841.05	0.000982808	0
DCTN2	-360.5	0.059511594	0.008470336
DCTN3	-280.55	0.137300243	0.026322017
DDX1	-671.85	0.001305221	0
DDX18	-1616.75	0.000982808	0
DDX39A	-1460.85	0.000982808	0
DDX39B	-988.85	0.000982808	0
DDX47	-1079.05	0.000982808	0

DDX5	-1571.35	0.000982808	0
DGUOK	-1480.75	0.000982808	0
DHRS7B	-252.1	0.180778349	0.038335543
DKC1	-1328.75	0.000982808	0
DKK1	-601.25	0.002684549	0
DLD	-1211.85	0.000982808	0
DLGAP5	-572.5	0.003808773	0
DMKN	-1623.35	0.000982808	0
DNAJA1	-1560.8	0.000982808	0
DNAJB6	-1396.05	0.000982808	0
DNAJC11	-340.15	0.074483341	0.01148073
DNAJC19	-1267.5	0.000982808	0
DNAJC8	-1466	0.000982808	0
DNMBP	10.85	0.965620571	0
DPH6	-396.4	0.039068264	0.00498452
DPY30	-1249.5	0.000982808	0
DSG2	82.95	0.68585166	0
DSTN	-979	0.000982808	0
DUSP14	-705.7	0.001101363	0
DUSP3	47.3	0.834129778	0
DYNLL1	-1739.05	0.000982808	0
DYNLRB1	-1111.75	0.000982808	0
DYNLT1	-1469.65	0.000982808	0
DYNLT3	-1506.1	0.000982808	0
EAF2	15	0.952906542	0
EBNA1BP2	-1327.25	0.000982808	0
EBP	-1400.95	0.000982808	0
ECH1	-616.05	0.002228938	0
ECHDC1	-832.75	0.000982808	0
EDF1	-1377.25	0.000982808	0
EED	-686.5	0.001200083	0
EEF1A1	-1753.05	0.000982808	0
EEF1B2	-1791.1	0.000982808	0
EEF1D	-1503.75	0.000982808	0
EEF1E1	-1486.3	0.000982808	0
EEF1G	-1805.1	0.000982808	0
EFEMP1	-766.75	0.00098324	0
EFNA1	-330.25	0.082394131	0.01326088
EIF1	-1749.65	0.000982808	0
EIF1AX	-1403.3	0.000982808	0
EIF2B3	-737.4	0.001001799	0
EIF2S1	-1590.45	0.000982808	0
EIF2S2	-1533	0.000982808	0
EIF3E	-1184.65	0.000982808	0
EIF3H	-1395.3	0.000982808	0
EIF3I	-1826.4	0.000982808	0
EIF3K	-1859.25	0.000982808	0
EIF3M	-1785.8	0.000982808	0

EIF4A1	-1244.45	0.000982808	0
EIF4A2	-1116.55	0.000982808	0
EIF4E2	-1370.4	0.000982808	0
EIF5	-1187.15	0.000982808	0
EIF5B	-1701.8	0.000982808	0
EMC3	-1563.7	0.000982808	0
EMC4	-1155.4	0.000982808	0
EMC7	-464.55	0.016322132	0.001412378
ENO1	-1911.55	0.000982808	0
ENY2	-1284.9	0.000982808	0
ERAL1	-484.4	0.012482143	0.001091209
ERGIC2	-1123	0.000982808	0
ERH	-1091.25	0.000982808	0
ESCO2	-678.95	0.001251828	0
ETFB	-722.85	0.001036939	0
EXOSC7	-849.9	0.000982808	0
EXOSC8	-1111.4	0.000982808	0
EXOSC9	-709.65	0.001086059	0
EZR	-1031.25	0.000982808	0
FABP5	-1781.55	0.000982808	0
FAM104B	-1009.75	0.000982808	0
FAM111A	-262.75	0.163544267	0.033498051
FAM111B	-770.55	0.00098313	0
FAM133B	-1312.05	0.000982808	0
FAM192A	-496.35	0.010594545	0.000750079
FAM32A	-1037.4	0.000982808	0
FAM60A	-1182.95	0.000982808	0
FAM96A	-1239	0.000982808	0
FANCD2	-422.5	0.028220517	0.003251284
FANCI	-514.2	0.008301993	0.000404178
FARSA	-237.35	0.207488423	0.045782706
FARSB	-1152.75	0.000982808	0
FASTKD2	-824.7	0.000982808	0
FAU	-1855.65	0.000982808	0
FDFT1	-1146.8	0.000982808	0
FDPS	-1339.05	0.000982808	0
FKBP14	-746.45	0.000999091	0
FKBP1A	-652.15	0.001524845	0
FKBP3	-1320.85	0.000982808	0
FTH1	-748.4	0.000995425	0
FTL	-2009.05	0.000982808	0
FTSJ2	-918.55	0.000982808	0
FUCA2	-231.9	0.217588491	0.04863599
G0S2	-341.3	0.073638412	0.011273954
G2E3	-336.05	0.077661785	0.012173906
G3BP1	-1095.8	0.000982808	0
GAPDH	-1731.4	0.000982808	0
GCC2	-253.85	0.177817036	0.037439608

GDI2	-750.1	0.000995409	0
GDPD1	79.35	0.702874504	0
GEMIN7	-924.95	0.000982808	0
GGCT	-1697.9	0.000982808	0
GHITM	-1425.55	0.000982808	0
GINS2	-917.25	0.000982808	0
GLO1	-1378.1	0.000982808	0
GLRX3	-419.1	0.029442513	0.003251284
GNB2L1	-1893.95	0.000982808	0
GNG10	-1360.85	0.000982808	0
GNG11	-833.4	0.000982808	0
GNG5	-1716.15	0.000982808	0
GNL3	-996.1	0.000982808	0
GORASP2	22.8	0.924390984	0
GOSR1	-1189.25	0.000982808	0
GPATCH1	-271.95	0.149288752	0.029665437
GPBP1	-621.6	0.002103781	0
GPX1	-605.65	0.002528376	0
GRPEL1	-835.75	0.000982808	0
GSTP1	-1901.95	0.000982808	0
GTF2A2	-1385.7	0.000982808	0
GTF3C6	-755.05	0.000990783	0
GYG1	-483.7	0.012588882	0.001091209
H2AFV	-1281.8	0.000982808	0
H2AFZ	-1906.35	0.000982808	0
H3F3A	-1475.25	0.000982808	0
H3F3B	-1740.15	0.000982808	0
HACD3	-1273.85	0.000982808	0
HADHA	-699.75	0.001137553	0
HARS2	-410.85	0.032713344	0.003837446
HAS2	-264.85	0.16052708	0.032875838
HAT1	-1234.35	0.000982808	0
HAUS1	-777.45	0.000983035	0
HAX1	-1029.2	0.000982808	0
HDDC2	-615.6	0.00224598	0
HES1	-839.15	0.000982808	0
HIGD1A	-1701.95	0.000982808	0
HILPDA	-859.9	0.000982808	0
HINFP	53	0.810762713	0
HINT1	-1914.8	0.000982808	0
HIST1H2BK	-584.9	0.003245645	0
HIST1H4C	-1506.95	0.000982808	0
HMGA2	15.35	0.95123796	0
HMGB1	-1959.05	0.000982808	0
HMGB2	-1131.7	0.000982808	0
HMGB3	-1579.25	0.000982808	0
HMGN1	-720.85	0.001046012	0
HMGN2	-1865.55	0.000982808	0

HMGN3	-1272.35	0.000982808	0
HMMR	-613.2	0.002307413	0
HMOX2	-511.35	0.008601364	0.000750079
HN1	-1394.55	0.000982808	0
HNRNPA1	-1938.95	0.000982808	0
HNRNPA2B1	-1615.7	0.000982808	0
HNRNPC	-1397.6	0.000982808	0
HNRNPF	-1261.65	0.000982808	0
HNRNPH2	-1091	0.000982808	0
HNRNPR	-1209.15	0.000982808	0
HOMER1	63.85	0.764839448	0
HSBP1	-1515.4	0.000982808	0
HSD17B10	-722.55	0.001036939	0
HSP90AA1	-1964.6	0.000982808	0
HSP90AB1	-1946.2	0.000982808	0
HSPA5	-986.15	0.000982808	0
HSPA8	-1913	0.000982808	0
HSPA9	-920.1	0.000982808	0
HSPD1	-1883.95	0.000982808	0
HUS1	-703.6	0.001117247	0
ID3	-669.05	0.001322967	0
IDI1	-708.25	0.001089974	0
IER3IP1	-1202.15	0.000982808	0
IFITM3	-1505.25	0.000982808	0
IFT22	-1047.65	0.000982808	0
IGBP1	-310.55	0.100957149	0.01734918
IL18	-811.5	0.000982808	0
IL1A	-249.9	0.184757484	0.039109618
IL1B	-544.6	0.005562173	0.000404178
IL32	-1501.75	0.000982808	0
IL7R	-387.35	0.04323574	0.005505267
ILF2	-1624.85	0.000982808	0
IMMP1L	-952	0.000982808	0
INHBA	-1781.05	0.000982808	0
INIP	-1111.25	0.000982808	0
INSIG2	-288.35	0.126636924	0.023688697
INTS7	-339.75	0.0748321	0.01148073
ITGB3BP	-932.35	0.000982808	0
JTB	-576.05	0.003639959	0
KARS	-692.1	0.001173478	0
KBTBD4	-366.3	0.055507059	0.007660839
KCTD9	11.15	0.965022344	0
KIAA0101	-1052.85	0.000982808	0
KIF23	-364.4	0.056755944	0.007932248
KPNA2	-950.35	0.000982808	0
KRT17	-431.25	0.025302898	0.002655394
KRT18	-1186.9	0.000982808	0
KRT19	-774.65	0.000983035	0

KRT6A	-369.85	0.053283684	0.007413248
KRT8	-1703.05	0.000982808	0
KTN1	-1568.95	0.000982808	0
LAMP2	-883	0.000982808	0
LAMTOR2	-1302.3	0.000982808	0
LAMTOR4	-1133.75	0.000982808	0
LAPTM4A	-1369.85	0.000982808	0
LARP4	-578.3	0.003528986	0
LBR	-512.7	0.008480398	0.000404178
LDHA	-1766.05	0.000982808	0
LDHB	-1796.35	0.000982808	0
LEO1	-1095.55	0.000982808	0
LGALS1	-837.75	0.000982808	0
LIMA1	-1065.4	0.000982808	0
LLPH	-1595.1	0.000982808	0
LRR1	-1213.85	0.000982808	0
LRRC63	13.8	0.957316454	0
LSM3	-1616.85	0.000982808	0
LSM5	-1916.95	0.000982808	0
LSM6	-1152.3	0.000982808	0
LUC7L3	-1062.05	0.000982808	0
LUZP1	-625.9	0.002020958	0
LUZP6	-1140.85	0.000982808	0
LYPLAL1	-833.75	0.000982808	0
LZTFL1	-236.3	0.209154592	0.046187188
MAD2L1	-1079.5	0.000982808	0
MAGOH	-1208.05	0.000982808	0
MAGOHB	-1467.15	0.000982808	0
MAPKAP1	93.35	0.64427106	0
MAPRE1	-1030.25	0.000982808	0
MATR3	-1256.85	0.000982808	0
MCFD2	-1032.15	0.000982808	0
MCTS1	-1487.05	0.000982808	0
MDH1	-1455.15	0.000982808	0
MDH2	-1532.6	0.000982808	0
ME2	2.35	0.991687455	0
MECR	-459.95	0.017335436	0.001739016
MED18	-302.1	0.110353028	0.019557065
MED27	-534.55	0.006370406	0.000404178
MED28	-953.1	0.000982808	0
MED6	-685.5	0.001203612	0
MED8	-689.6	0.001185892	0
METTL3	-707.2	0.00109351	0
METTL4	75.55	0.716138118	0
METTL5	-1363.6	0.000982808	0
MFN1	-235.45	0.210737313	0.046606128
MGME1	55.2	0.801046811	0
MGST1	-1600.15	0.000982808	0

MGST3	-959.35	0.000982808	0
MIF	-769.65	0.00098313	0
MIR205HG	-1637.55	0.000982808	0
MKKS	-1021.25	0.000982808	0
MLLT11	-826.25	0.000982808	0
MMP1	-318.45	0.092740595	0.015642735
MOB1A	-332.7	0.080449523	0.012795205
MOCS3	51.1	0.818497271	0
MORF4L1	-857.7	0.000982808	0
MORF4L2	-1658.7	0.000982808	0
MPHOSPH10	-1146.9	0.000982808	0
MPHOSPH6	-1250.35	0.000982808	0
MRPL13	-1677.8	0.000982808	0
MRPL14	-910.35	0.000982808	0
MRPL16	-536.95	0.006170006	0.000404178
MRPL18	-1309.05	0.000982808	0
MRPL20	-1720.85	0.000982808	0
MRPL21	-1657.25	0.000982808	0
MRPL22	-1591.15	0.000982808	0
MRPL27	-1383.8	0.000982808	0
MRPL3	-813.65	0.000982808	0
MRPL32	-1909.3	0.000982808	0
MRPL33	-1579.35	0.000982808	0
MRPL36	-1131.9	0.000982808	0
MRPL39	-1361.6	0.000982808	0
MRPL42	-1085.15	0.000982808	0
MRPL50	-1526.5	0.000982808	0
MRPL51	-1796.6	0.000982808	0
MRPL52	-1170.7	0.000982808	0
MRPL55	-642.45	0.00168147	0
MRPS10	-601.9	0.002662966	0
MRPS11	-411.55	0.032435288	0.003837446
MRPS15	-1006.15	0.000982808	0
MRPS17	-1711.45	0.000982808	0
MRPS18C	-1595.3	0.000982808	0
MRPS21	-1471.75	0.000982808	0
MRPS23	-1320.5	0.000982808	0
MRPS24	-1187.3	0.000982808	0
MRPS33	-1759.35	0.000982808	0
MRPS35	-890.25	0.000982808	0
MRPS9	-1162.7	0.000982808	0
MRTO4	-1203.4	0.000982808	0
MSH2	-413.7	0.031576923	0.00354774
MSL3	-884.7	0.000982808	0
MT1E	-430.3	0.02559673	0.002655394
MT1X	-1382	0.000982808	0
MT2A	-1951.05	0.000982808	0
MTCH2	-1465.05	0.000982808	0

MTF2	-402.35	0.036363352	0.00440466
MTHFD2	-570.75	0.003879604	0
MTRNR2L1	-1551.65	0.000982808	0
MTRNR2L2	-1972.2	0.000982808	0
MTRNR2L8	-1970.75	0.000982808	0
MTRNR2L9	-1607.95	0.000982808	0
MYEOV2	-1199.4	0.000982808	0
MYL12A	-1655.6	0.000982808	0
MYL12B	-1839.2	0.000982808	0
MYL6	-1970.2	0.000982808	0
MZT1	-645.2	0.001632025	0
NAA20	-1500.3	0.000982808	0
NACA	-1971.85	0.000982808	0
NADK2	16.3	0.948394717	0
NAE1	-855.65	0.000982808	0
NANS	-508.95	0.008897222	0.000750079
NBN	-275.95	0.143517435	0.028092356
NCL	-1105.1	0.000982808	0
NCOA7	-442.4	0.021835552	0.0023441
NDC80	-540.8	0.005859792	0.000404178
NDUFA1	-1929.35	0.000982808	0
NDUFA11	-1371.7	0.000982808	0
NDUFA12	-1435.9	0.000982808	0
NDUFA13	-1900.95	0.000982808	0
NDUFA2	-1546.55	0.000982808	0
NDUFA3	-1563.85	0.000982808	0
NDUFA4	-2031.2	0.000982808	0
NDUFA8	-1245.55	0.000982808	0
NDUFA9	-1315.75	0.000982808	0
NDUFAB1	-1006.4	0.000982808	0
NDUFAF4	-843.1	0.000982808	0
NDUFB11	-1342.55	0.000982808	0
NDUFB2	-1989.45	0.000982808	0
NDUFB3	-1497.9	0.000982808	0
NDUFB4	-1888.4	0.000982808	0
NDUFB5	-1111.5	0.000982808	0
NDUFB6	-1733.4	0.000982808	0
NDUFB7	-477.3	0.013752207	0.001091209
NDUFB8	-1530.3	0.000982808	0
NDUFB9	-1779.05	0.000982808	0
NDUFC1	-1196.15	0.000982808	0
NDUFS2	-567.45	0.004058005	0
NDUFS3	-551.1	0.005069022	0.000404178
NDUFS5	-1924.3	0.000982808	0
NDUFS6	-693.35	0.001171273	0
NDUFS7	-601.8	0.002666428	0
NDUFS8	-1350.3	0.000982808	0
NDUFV1	-414.65	0.031205101	0.00354774

NDUFV2	-1433.45	0.000982808	0
NEK3	28.65	0.905426728	0
NEXN	-487.9	0.01185693	0.001091209
NHP2	-1469.4	0.000982808	0
NIFK	-1209.7	0.000982808	0
NIP7	-1041.45	0.000982808	0
NME2	-1665.55	0.000982808	0
NMI	-576.25	0.003626979	0
NOL12	-265.85	0.158920763	0.032224756
NOP10	-1857.45	0.000982808	0
NOP16	-1577.65	0.000982808	0
NOP58	-1305.65	0.000982808	0
NPC2	-1700.05	0.000982808	0
NPM1	-1950.45	0.000982808	0
NQO1	-1109.3	0.000982808	0
NR2C2AP	-631.35	0.001903774	0
NRDC	-444.7	0.021210894	0.002035205
NSFL1C	-411.75	0.032360729	0.003837446
NSMCE2	-360.6	0.059460367	0.008470336
NSRP1	-1459.95	0.000982808	0
NTMT1	-983.75	0.000982808	0
NUDC	-458.2	0.01773913	0.001739016
NUDT1	-817.1	0.000982808	0
NUDT5	-1217.2	0.000982808	0
NUF2	-491.45	0.011317279	0.001091209
NUPL2	-744.35	0.000999091	0
NUSAP1	-995.5	0.000982808	0
NUTF2	-1409.8	0.000982808	0
NXT2	-239.45	0.203840733	0.044628361
OAZ1	-965.2	0.000982808	0
OCIAD2	-1647.7	0.000982808	0
ODC1	-779.9	0.000983035	0
ODF2L	-1100.4	0.000982808	0
OGFOD1	-397.05	0.038793086	0.00498452
OLA1	-1407.15	0.000982808	0
ORC5	-681.75	0.001235294	0
ORMDL1	-792.95	0.000982808	0
ORMDL2	-883.55	0.000982808	0
OSER1	-916.5	0.000982808	0
OSGEP	-446.45	0.020746076	0.002035205
OSGIN2	-262.75	0.163544267	0.033498051
OST4	-1926.65	0.000982808	0
OSTC	-1451	0.000982808	0
OTUB1	-859.15	0.000982808	0
PA2G4	-1747.2	0.000982808	0
PABPC1	-1547.55	0.000982808	0
PAFAH1B3	-487.3	0.011958507	0.001091209
PAICS	-1129.5	0.000982808	0

PAIP2	-1088.3	0.000982808	0
PARK7	-1731.5	0.000982808	0
PBDC1	-1362.9	0.000982808	0
PBK	-562.65	0.004356419	0
PCID2	-697.5	0.001154882	0
PCNA	-1146.75	0.000982808	0
PCNP	-835.25	0.000982808	0
PDCD10	-1133.8	0.000982808	0
PDCD5	-1723.05	0.000982808	0
PDCD6	-365.05	0.05634378	0.007932248
PDCL3	-571.5	0.003851675	0
PDE4DIP	71.8	0.732552098	0
PDHA1	-1246.55	0.000982808	0
PDIA3	-494.7	0.010832225	0.000750079
PDIA6	-1001.85	0.000982808	0
PDZD11	-1081.15	0.000982808	0
PEA15	-234.55	0.212366533	0.047057123
PEBP1	-871.55	0.000982808	0
PET100	-1564.85	0.000982808	0
PFDN1	-1372	0.000982808	0
PFDN4	-1353.85	0.000982808	0
PFDN5	-1864.05	0.000982808	0
PFN1	-888.55	0.000982808	0
PGAM1	-488	0.011846085	0.001091209
PGK1	-1671.65	0.000982808	0
PGM3	-538.35	0.006054439	0.000404178
PHB	-1636.9	0.000982808	0
PHPT1	-1582.4	0.000982808	0
PIAS2	25.6	0.917383785	0
PIGK	-306.75	0.105139957	0.018411743
PIGP	-385.25	0.044369401	0.005775985
PKM	-1230.1	0.000982808	0
PLAU	-528.95	0.006850989	0.000404178
PLAUR	-1355.85	0.000982808	0
PLIN2	-598.95	0.002745733	0
PLP2	-1063.45	0.000982808	0
PLRG1	-637.45	0.001788872	0
PLS3	-1199.1	0.000982808	0
PLSCR1	-1024.55	0.000982808	0
PMPCB	-1063.25	0.000982808	0
PNPLA4	-839.55	0.000982808	0
PNPLA8	-405.7	0.034800261	0.004126617
POLD2	-554	0.004882216	0.000404178
POLR1C	-555.9	0.004766845	0.000404178
POLR1E	-517	0.008029117	0.000404178
POLR2F	-1385.95	0.000982808	0
POLR2G	-1182.9	0.000982808	0
POLR2H	-1231.9	0.000982808	0

POLR2I	-1731.25	0.000982808	0
POLR2J	-1231.45	0.000982808	0
POLR2K	-1079.15	0.000982808	0
POLR2L	-1757.65	0.000982808	0
POLR3GL	99.35	0.619560571	0
POLR3K	-1369.35	0.000982808	0
POMP	-1962.7	0.000982808	0
POP4	-833.5	0.000982808	0
PPA1	-1166.3	0.000982808	0
PPA2	-386.95	0.043463169	0.005505267
PPHLN1	-1176.5	0.000982808	0
PPIA	-2041.3	0.000982808	0
PPIB	-1294.4	0.000982808	0
PPIL1	-424.1	0.027651916	0.002953206
PPIL4	-690.3	0.001184385	0
PPP1CA	-794.35	0.000982808	0
PPP1R7	-711.5	0.001080242	0
PPP2R3C	-508.1	0.009006786	0.000750079
PPP2R5C	-869.05	0.000982808	0
PPP6C	-241.45	0.199889157	0.04365059
PQBP1	-890.75	0.000982808	0
PRDX1	-1931.7	0.000982808	0
PRDX2	-1692.6	0.000982808	0
PRDX3	-924.25	0.000982808	0
PRDX4	-1294.6	0.000982808	0
PRDX5	-1048.8	0.000982808	0
PRDX6	-1520.4	0.000982808	0
PRELID1	-598.55	0.002758706	0
PRELID3B	-793.05	0.000982808	0
PRKDC	-543.25	0.005652742	0.000404178
PRMT1	-867.05	0.000982808	0
PRSS23	-337.65	0.076426667	0.011963048
PSENEN	-1265.85	0.000982808	0
PSMA1	-1637.9	0.000982808	0
PSMA2	-1987.9	0.000982808	0
PSMA3	-1717.5	0.000982808	0
PSMA4	-1778.8	0.000982808	0
PSMA5	-1626.2	0.000982808	0
PSMA6	-1717.6	0.000982808	0
PSMA7	-1546.15	0.000982808	0
PSMB1	-1863.3	0.000982808	0
PSMB2	-1753.85	0.000982808	0
PSMB3	-1792.1	0.000982808	0
PSMB4	-1266.75	0.000982808	0
PSMB5	-1711.95	0.000982808	0
PSMB6	-1589.45	0.000982808	0
PSMB7	-1837.35	0.000982808	0
PSMC1	-1823.9	0.000982808	0

PSMC2	-1788.45	0.000982808	0
PSMC3IP	-659.45	0.001423137	0
PSMC4	-1428	0.000982808	0
PSMD10	-1064.8	0.000982808	0
PSMD11	-1595.35	0.000982808	0
PSMD12	-855.95	0.000982808	0
PSMD14	-1536.7	0.000982808	0
PSMD2	-729	0.001014197	0
PSMD4	-1248.75	0.000982808	0
PSMD6	-793.7	0.000982808	0
PSME1	-897.15	0.000982808	0
PSME2	-1318.2	0.000982808	0
PSME3	-545.1	0.005529508	0.000404178
PSMG1	-739.05	0.001001799	0
PSMG2	-1015.05	0.000982808	0
PTGES3	-1334.9	0.000982808	0
PTGR1	-1088.15	0.000982808	0
PTHLH	-703.15	0.001118846	0
PTMA	-2002.5	0.000982808	0
PTN	-1539.45	0.000982808	0
PTP4A2	-604.35	0.002573171	0
PTPRA	127.4	0.510286285	0
PTPRK	37.7	0.869282542	0
PTRH2	-792.95	0.000982808	0
PTTG1	-1560.45	0.000982808	0
PUF60	-792.25	0.000982808	0
PUM3	-1514.9	0.000982808	0
PWP1	-1132.15	0.000982808	0
QPCT	-354.65	0.063719828	0.009288133
RAB11A	-758.95	0.000986611	0
RAB3IP	-232.5	0.216231619	0.048231025
RABEPK	-1278.85	0.000982808	0
RABGGTB	-1085.6	0.000982808	0
RABL3	-758.5	0.000986611	0
RALA	-443.25	0.021607341	0.002035205
RALB	-511	0.008636927	0.000750079
RAN	-1891.55	0.000982808	0
RAP1A	-438.95	0.022806229	0.0023441
RARS	-1325.5	0.000982808	0
RBBP7	-832.15	0.000982808	0
RBFA	63.1	0.767480388	0
RBM25	-523.8	0.007350538	0.000404178
RBM3	-1895.45	0.000982808	0
RBM39	-1713.25	0.000982808	0
RBM4	-658.05	0.001440579	0
RBM48	-571.6	0.003848495	0
RBM8A	-1812.65	0.000982808	0
RBMX	-1862.2	0.000982808	0

RBX1	-954.85	0.000982808	0
RCC1	-1215.35	0.000982808	0
RDH11	-837.4	0.000982808	0
RFC4	-1186.6	0.000982808	0
RFXANK	-860	0.000982808	0
RHOA	-1456.85	0.000982808	0
RNASEH2A	-523.7	0.007351044	0.000404178
RNA_SPIKE_ERCC	29.65	0.900785129	0
RNF181	-1253.35	0.000982808	0
RNF34	-625.8	0.002021316	0
ROMO1	-1765.55	0.000982808	0
RPA3	-1931.15	0.000982808	0
RPAP3	-794.65	0.000982808	0
RPF2	-1371.55	0.000982808	0
RPL10	-1951.35	0.000982808	0
RPL10A	-1927.1	0.000982808	0
RPL11	-1889.85	0.000982808	0
RPL12	-1824.75	0.000982808	0
RPL13	-796.9	0.000982808	0
RPL13A	-1979.05	0.000982808	0
RPL14	-1922.05	0.000982808	0
RPL15	-1930.2	0.000982808	0
RPL18	-1917.8	0.000982808	0
RPL18A	-1808.05	0.000982808	0
RPL19	-1965.15	0.000982808	0
RPL21	-1992.05	0.000982808	0
RPL22	-1836.75	0.000982808	0
RPL23	-1923.1	0.000982808	0
RPL23A	-2006.7	0.000982808	0
RPL24	-1960.85	0.000982808	0
RPL26	-1935.65	0.000982808	0
RPL26L1	-1671.2	0.000982808	0
RPL27	-1804.6	0.000982808	0
RPL27A	-1860.9	0.000982808	0
RPL28	-1695.85	0.000982808	0
RPL29	-1827.85	0.000982808	0
RPL3	-1673.2	0.000982808	0
RPL30	-1923.65	0.000982808	0
RPL31	-1982.55	0.000982808	0
RPL32	-2026.1	0.000982808	0
RPL34	-1998.7	0.000982808	0
RPL35	-2011.8	0.000982808	0
RPL35A	-1921.25	0.000982808	0
RPL36	-1980.3	0.000982808	0
RPL36AL	-1842.45	0.000982808	0
RPL37	-1889.55	0.000982808	0
RPL37A	-2006.55	0.000982808	0
RPL38	-1996.5	0.000982808	0

RPL39	-2023.3	0.000982808	0
RPL4	-1757.55	0.000982808	0
RPL41	-2010.55	0.000982808	0
RPL5	-1954.65	0.000982808	0
RPL6	-1932.35	0.000982808	0
RPL7	-1705.35	0.000982808	0
RPL7A	-1968.8	0.000982808	0
RPL8	-1792.1	0.000982808	0
RPL9	-1949.65	0.000982808	0
RPLP0	-1785.15	0.000982808	0
RPLP1	-1940.9	0.000982808	0
RPLP2	-1988.1	0.000982808	0
RPN2	-1252.85	0.000982808	0
RPP21	-1156.9	0.000982808	0
RPP30	-1035	0.000982808	0
RPP40	-299.75	0.113311022	0.020344933
RPS11	-1975.45	0.000982808	0
RPS12	-1918.9	0.000982808	0
RPS13	-1991.4	0.000982808	0
RPS14	-1926.7	0.000982808	0
RPS15	-1604.35	0.000982808	0
RPS15A	-1819.85	0.000982808	0
RPS16	-2018.85	0.000982808	0
RPS18	-1965.25	0.000982808	0
RPS19	-2041.55	0.000982808	0
RPS2	-1518.95	0.000982808	0
RPS20	-1689.35	0.000982808	0
RPS21	-1975.4	0.000982808	0
RPS23	-2016.5	0.000982808	0
RPS24	-1970.4	0.000982808	0
RPS25	-1904.45	0.000982808	0
RPS26	-2032.7	0.000982808	0
RPS27	-1998.7	0.000982808	0
RPS27A	-1951.05	0.000982808	0
RPS28	-1962	0.000982808	0
RPS29	-1939.9	0.000982808	0
RPS3	-1982.75	0.000982808	0
RPS3A	-1979.5	0.000982808	0
RPS4X	-1948.15	0.000982808	0
RPS5	-1935.95	0.000982808	0
RPS6	-1991.95	0.000982808	0
RPS7	-1875.6	0.000982808	0
RPS8	-1916.4	0.000982808	0
RPS9	-1429	0.000982808	0
RQCD1	-357.65	0.061573112	0.009006543
RRM1	-1148.85	0.000982808	0
RRP15	-1103.5	0.000982808	0
RSL1D1	-1215.95	0.000982808	0

RSL24D1	-954.1	0.000982808	0
RSRC1	-1205.3	0.000982808	0
RSRC2	-843.35	0.000982808	0
RTCB	-1061.5	0.000982808	0
RTFDC1	-911.1	0.000982808	0
RUVBL1	-993.9	0.000982808	0
RUVBL2	-382.1	0.04609364	0.006055578
RWDD1	-1396.35	0.000982808	0
S100A10	-1784.7	0.000982808	0
S100A11	-1957.95	0.000982808	0
S100A13	-1229.15	0.000982808	0
S100A2	-1977.8	0.000982808	0
S100A6	-1679.95	0.000982808	0
SARNP	-1443.5	0.000982808	0
SARS	-1216.15	0.000982808	0
SAT1	-1454.25	0.000982808	0
SC5D	-396.7	0.038961578	0.00498452
SCFD1	-889.7	0.000982808	0
SCNM1	-762.55	0.000986059	0
SDF2	-936.45	0.000982808	0
SDHD	-920.2	0.000982808	0
SEC13	-1671.4	0.000982808	0
SEC61B	-1701.95	0.000982808	0
SEC61G	-1996.15	0.000982808	0
SEC62	-1298	0.000982808	0
SEH1L	-408.7	0.033596427	0.004126617
SELT	-919.6	0.000982808	0
15/Sep	-1130.65	0.000982808	0
SEPW1	-1491.5	0.000982808	0
SERF2	-1835.1	0.000982808	0
SERPINB2	-952.75	0.000982808	0
SET	-1510.75	0.000982808	0
SETD5	-439.4	0.022678532	0.0023441
SF3A3	-1002.7	0.000982808	0
SF3B6	-1708.95	0.000982808	0
SFT2D1	-536.95	0.006170006	0.000404178
SGCB	-395.1	0.039645427	0.00498452
SH2D4A	-501.95	0.009824034	0.000750079
SH3BGRL3	-1344.1	0.000982808	0
SHFM1	-2028.05	0.000982808	0
SIVA1	-831.15	0.000982808	0
SKA2	-1140.25	0.000982808	0
SKP1	-1872.9	0.000982808	0
SLC25A26	-400.85	0.037075364	0.004709457
SLC25A39	-298.3	0.114951368	0.020940308
SLC25A5	-2011.6	0.000982808	0
SLC39A4	-499.6	0.010174219	0.000750079
SLC43A3	-261.45	0.165549378	0.033983234

SLC50A1	-329.5	0.082938119	0.013373352
SLIRP	-1958.9	0.000982808	0
SMARCE1	-1095.85	0.000982808	0
SMC2	-998.35	0.000982808	0
SMC4	-444.5	0.021263393	0.002035205
SMS	-1282.4	0.000982808	0
SMUG1	-714.4	0.001075521	0
SNRPA1	-1215.6	0.000982808	0
SNRPB	-716.75	0.001065066	0
SNRPB2	-1546.1	0.000982808	0
SNRPC	-1267.8	0.000982808	0
SNRPD1	-1723.85	0.000982808	0
SNRPD2	-2033.35	0.000982808	0
SNRPD3	-1338.2	0.000982808	0
SNRPE	-1929.75	0.000982808	0
SNRPF	-1892.5	0.000982808	0
SNRPG	-1973.35	0.000982808	0
SNRPN	63.15	0.767383151	0
SNW1	-1307.25	0.000982808	0
SNX3	-338.95	0.075326267	0.01172267
SOD1	-1711	0.000982808	0
SON	-1761.65	0.000982808	0
SOX4	-1789.3	0.000982808	0
SPC25	-855.2	0.000982808	0
SPCS2	-1322.3	0.000982808	0
SPINT2	-797.85	0.000982808	0
SRFBP1	-420.55	0.028915459	0.003251284
SRGAP1	-442.85	0.021716944	0.002035205
SRI	-1532	0.000982808	0
SRP14	-1960.7	0.000982808	0
SRP54	-852.55	0.000982808	0
SRP72	-570.4	0.003888548	0
SRP9	-1499.55	0.000982808	0
SRRM1	-1518.4	0.000982808	0
SRSF10	-1285.8	0.000982808	0
SRSF3	-1795.2	0.000982808	0
SRSF5	-888.3	0.000982808	0
SRSF7	-1601	0.000982808	0
SSB	-1635.75	0.000982808	0
SSBP1	-1985.35	0.000982808	0
SSR2	-910.55	0.000982808	0
SSR3	-1329.7	0.000982808	0
SSR4	-1658.5	0.000982808	0
SSSCA1	-789.2	0.000982808	0
STMN1	-1757.2	0.000982808	0
STOML2	-994.7	0.000982808	0
STRAP	-1285.95	0.000982808	0
STRBP	-450.1	0.0196991	0.001739016

STX17	-382.2	0.046052104	0.006055578
STYXL1	-586.35	0.003180042	0
SUB1	-1625.1	0.000982808	0
SUGT1	-950.95	0.000982808	0
SUMO1	-1470	0.000982808	0
SUMO2	-1859.15	0.000982808	0
SUPT4H1	-410.6	0.032796365	0.003837446
SURF4	-389.25	0.04231231	0.005505267
SYNCRIP	-1077.55	0.000982808	0
SYPL1	-1242.05	0.000982808	0
SYTL4	-336.9	0.076927556	0.011963048
TAF7	-1036.15	0.000982808	0
TALDO1	-1057.75	0.000982808	0
TARDBP	-993.4	0.000982808	0
TAX1BP1	-1500.95	0.000982808	0
TBC1D15	-325.4	0.086221457	0.014014648
TBCA	-1494.1	0.000982808	0
TBRG4	-342.1	0.073073824	0.011273954
TCEAL8	-1433.3	0.000982808	0
TCEAL9	-1702.15	0.000982808	0
TCEB1	-1295.35	0.000982808	0
TCEB2	-1628.55	0.000982808	0
TCP1	-1716.65	0.000982808	0
TEFM	-345.25	0.070741436	0.010785823
TFG	-328.8	0.083469241	0.013584836
THG1L	-400.15	0.03735718	0.004709457
THOC1	-232.5	0.216231619	0.048231025
THOC7	-810.7	0.000982808	0
THRAP3	-1273.7	0.000982808	0
THUMPD3	-1581.6	0.000982808	0
THYN1	-768.8	0.00098324	0
TIMM10	-1491.4	0.000982808	0
TIMM13	-1445.95	0.000982808	0
TIMM17A	-1590.7	0.000982808	0
TIMM21	-865.45	0.000982808	0
TIMM22	-255.1	0.175868199	0.036973682
TIMM8A	-974.35	0.000982808	0
TIMMDC1	-740.4	0.001001799	0
TK1	-425.9	0.027022174	0.002953206
TMA7	-1884	0.000982808	0
TMBIM6	-1823.45	0.000982808	0
TMCO1	-1374.75	0.000982808	0
TMED10	-1208.4	0.000982808	0
TMEM106C	-976.75	0.000982808	0
TMEM120A	42.45	0.850043217	0
TMEM123	50.65	0.820278453	0
TMEM126A	-977.15	0.000982808	0
TMEM14C	-998.2	0.000982808	0

TMEM167A	-1277.2	0.000982808	0
TMEM179B	-476.95	0.013804527	0.001091209
TMEM208	-1091.55	0.000982808	0
TMEM216	-328.45	0.083718386	0.013584836
TMEM258	-1884.3	0.000982808	0
TMEM50A	-870.6	0.000982808	0
TMEM60	-687.65	0.001192721	0
TMEM97	-898.05	0.000982808	0
TMOD3	-317.65	0.093560725	0.015859091
TMSB10	-1978.3	0.000982808	0
TMSB15A	-948.5	0.000982808	0
TMSB15B	-1246.7	0.000982808	0
TMSB4X	-2034.25	0.000982808	0
TMX1	-1152.15	0.000982808	0
TNFAIP8	21.6	0.927685118	0
TNFRSF12A	-241.2	0.200395694	0.043859779
TOMM22	-1044.25	0.000982808	0
TOMM5	-1850.3	0.000982808	0
TOMM6	-1627.6	0.000982808	0
TOMM7	-1948.6	0.000982808	0
TOP2A	-924.55	0.000982808	0
TPGS2	-420.25	0.029002681	0.003251284
TPI1	-1833.7	0.000982808	0
TPM1	-788.15	0.000982808	0
TPM3	-1217.2	0.000982808	0
TPRKB	-1421	0.000982808	0
TPT1	-1898.4	0.000982808	0
TPX2	-327.45	0.084527492	0.013812997
TRAPPC2	-289.9	0.124776517	0.023276554
TRAPPC3	-419.4	0.02932611	0.003251284
TRIP10	58	0.788123992	0
TRMT10C	-821.4	0.000982808	0
TRMT112	-1910.55	0.000982808	0
TRPM7	47.75	0.832443688	0
TRUB2	-395.5	0.039480957	0.00498452
TSFM	-818.6	0.000982808	0
TSG101	-326	0.085828877	0.013886094
TSPAN6	-1282.8	0.000982808	0
TSR2	-758.45	0.000986611	0
TSTA3	-370.85	0.052682711	0.007268555
TTC26	-389.9	0.042014721	0.005266146
TUBA1B	-2010.9	0.000982808	0
TUBA1C	-1557.75	0.000982808	0
TUBA4A	-1303.3	0.000982808	0
TUBB	-1360.3	0.000982808	0
TUBB4B	-1067.75	0.000982808	0
TWISTNB	-1239.4	0.000982808	0
TXN	-2027.95	0.000982808	0

TXNDC17	-1574	0.000982808	0
U2SURP	-1822.1	0.000982808	0
UBA3	-773.55	0.000983035	0
UBA52	-2028.3	0.000982808	0
UBAP2	-285.4	0.130627607	0.024554213
UBB	-1796.3	0.000982808	0
UBE2C	-561.5	0.004434504	0
UBE2D3	-1022.4	0.000982808	0
UBE2E2	-235.65	0.21033309	0.046435429
UBE2N	-1374.25	0.000982808	0
UBE2T	-941.2	0.000982808	0
UBE2V2	-1218.85	0.000982808	0
UBL5	-1820.95	0.000982808	0
UBXN1	-512.45	0.008504353	0.000562909
UBXN4	-708.15	0.001089974	0
UCHL3	-1261.6	0.000982808	0
UFC1	-929.9	0.000982808	0
UFM1	-1048.45	0.000982808	0
UGP2	-845.55	0.000982808	0
UGT1A6	-919.8	0.000982808	0
UNC50	-460.9	0.017153713	0.001739016
UQCC2	-1096.8	0.000982808	0
UQCR10	-1546.5	0.000982808	0
UQCR11	-1481.25	0.000982808	0
UQCRB	-1789.55	0.000982808	0
UQCRC1	-822	0.000982808	0
UQCRC2	-387.8	0.043001768	0.005505267
UQCRFS1	-276.65	0.142546166	0.027794738
UQCRH	-1938.65	0.000982808	0
UQCRQ	-1857.55	0.000982808	0
UROD	-878.75	0.000982808	0
USE1	-323.35	0.088111136	0.014437604
USMG5	-1899.8	0.000982808	0
USP15	-511.65	0.008578212	0.000750079
USP16	-915.2	0.000982808	0
UTP11	-1164.55	0.000982808	0
UTP6	-830.65	0.000982808	0
UXT	-916.5	0.000982808	0
VAMP8	-1106.6	0.000982808	0
VBP1	-1502.45	0.000982808	0
VDAC3	-1108.5	0.000982808	0
VMP1	-684.1	0.001214403	0
VPS26A	-877.4	0.000982808	0
VPS29	-1063.8	0.000982808	0
VPS35	-876.15	0.000982808	0
VPS41	-328.5	0.083707492	0.013584836
VRK1	-668.25	0.001328822	0
VTA1	-1078.6	0.000982808	0

WARS	-522.2	0.007508833	0.000404178
WBSCR22	-1496.75	0.000982808	0
WDHD1	-323.3	0.088111136	0.014437604
WDR3	-237.45	0.207377529	0.045704871
WDR43	-647.25	0.00159946	0
WDR83OS	-1516.7	0.000982808	0
WDR92	-477.95	0.013627797	0.001091209
XRCC5	-1557.1	0.000982808	0
XRCC6	-1025.95	0.000982808	0
YIPF5	-982.75	0.000982808	0
YIPF6	-731.7	0.001013321	0
YWHAB	-1282.4	0.000982808	0
YWHAE	-1685.2	0.000982808	0
YWHAQ	-607.75	0.002458603	0
YWHAZ	-1443.95	0.000982808	0
ZC3H10	-1984.2	0.000982808	0
ZCRB1	-1246.75	0.000982808	0
ZFAND1	-408.35	0.03372231	0.004126617
ZMPSTE24	-901.3	0.000982808	0
ZNF277	-349.55	0.067555951	0.010057307
ZNF326	-713.7	0.001076756	0
ZNF530	-266.1	0.158490755	0.032224756
ZNF670	-248.45	0.187205549	0.039763221
ZNF691	-256.4	0.173876828	0.036327786
ZNF714	-276.65	0.142546166	0.027794738
ZNF81	-298.6	0.114671374	0.020821435
ZNHIT3	-832.35	0.000982808	0
ZNRD1	-507.4	0.009092672	0.000750079
ZRANB2	-1402.05	0.000982808	0
ZWINT	-701.5	0.001132316	0

TABLE S3: A total of 334 differentially expressed (DE) genes between cell populations of 5637PR vs. stressed 5637PR.

See below

Table S3. A total of 334 differentially expressed (DE) genes between cell populations of 5637PR vs. stressed 5637PR.

Gene symbol	DE score	*p* value	*q* value
ACTB	421.3	0.07043913	0.01303284
ACTG1	336.5	0.148075452	0.037211197
ACYP1	808.75	0.013996154	0
ADSL	332.75	0.152776915	0.038676348
ANKRD1	-103.55	0.700880435	0
ANXA2	-173.5	0.474920396	0
ARL6IP1	-297.65	0.204934462	0
ASF1B	336.1	0.148245772	0.037211197
ATP5A1	685.9	0.013996154	0
ATP5E	359.95	0.122797181	0.029065631
ATP5L	419.6	0.071272021	0.013991269
ATP6V0B	760.4	0.013996154	0
AURKA	348.9	0.134321101	0.032595691
B2M	439.7	0.059265166	0.010220625
BANF1	374.25	0.107394459	0.024577201
BCAP31	502.15	0.031453652	0.003979921
BEX3	385.4	0.097278955	0.021895171
BRIX1	657.9	0.013996154	0
BRK1	361.4	0.121930435	0.028613746
BSG	398.5	0.085500754	0.017891608
BTF3	403.9	0.082125	0.016476493
C10orf88	-24.15	0.943088057	0
C11orf58	549.25	0.022286008	0.002427099
C15orf48	490.35	0.035895778	0.00537723
C5orf30	-26.1	0.937548633	0
C9orf78	508.95	0.029850592	0.003979921
CACYBP	671.75	0.013996154	0
CALM2	493.15	0.034577333	0.003979921
CALU	408.5	0.078389871	0.015597162
CAV1	-33.45	0.919472879	0
CCNB1	-40.15	0.902012793	0
CCNB2	324.2	0.165342342	0.042707613
CCT2	475.2	0.041359524	0.006729685
CCT3	467.1	0.044730159	0.006729685
CCT5	508.9	0.029850592	0.003979921
CCT7	598.35	0.017037975	0
CCT8	582.75	0.018405028	0
CD47	-13.4	0.968220875	0
CD59	522.6	0.026864238	0.002427099
CDC45	339.3	0.144837297	0.035597457
CDC5L	435.4	0.061796756	0.011237424
CDC6	697.2	0.013996154	0
CDC73	-67	0.819722536	0
CDCA4	-84.8	0.760871142	0
CDK1	698	0.013996154	0

CDKN3	-45.95	0.885031209	0
CKS1B	500.55	0.031945833	0.003979921
CLIC1	474.25	0.041613208	0.006729685
CLSPN	888.35	0.013996154	0
CNIH4	312.25	0.182520677	0.048553522
COMMD4	626.55	0.014333333	0
COPS8	353.15	0.12998408	0.030625633
COX4I1	579.85	0.018914365	0
COX7B	-36.4	0.91220202	0
COX8A	515.6	0.028699686	0.003979921
CSF2	586.05	0.017979769	0
CSTB	334.4	0.150276963	0.037521367
CTSC	605.8	0.015953642	0
CTSV	510	0.029734234	0.003979921
CXCL8	802.7	0.013996154	0
DDX19A	346.85	0.136790011	0.033108196
DDX5	331.45	0.154237358	0.039022336
DEPDC1	-144.15	0.565671801	0
DNAJA1	904.95	0.013996154	0
DPY30	449.2	0.053775154	0.009139078
DYNLL1	455.1	0.050830851	0.007925753
DYNLT1	316	0.177815739	0.046535053
EBNA1BP2	548.95	0.022286008	0.002427099
EDEM1	-42.65	0.893911255	0
EDF1	630.5	0.013996154	0
EED	360	0.122797181	0.029065631
EEF1A1	717	0.013996154	0
EEF1B2	685.6	0.013996154	0
EEF1D	555.9	0.022286008	0
EEF1G	536.05	0.024516791	0.002427099
EIF1	795.65	0.013996154	0
EIF2S1	579.4	0.018914835	0
EIF3E	-28.35	0.93377327	0
EIF3I	607.05	0.015953642	0
EIF3M	379.9	0.102023161	0.022339968
EIF4A2	337.95	0.146326367	0.036694185
EIF4E2	383.8	0.098694832	0.021895171
EMC3	546.45	0.022426587	0.002427099
ENKUR	-69.85	0.808731678	0
ENO1	564.05	0.021014563	0
ESCO2	656.45	0.013996154	0
FAM96A	437.85	0.060570874	0.011237424
FANCD2	412.85	0.075968439	0.014805306
FANCI	327.25	0.160697462	0.040957301
FAU	822.1	0.013996154	0
FTH1	429.95	0.06521875	0.012202175
FTL	915.4	0.013996154	0
GAPDH	826.05	0.013996154	0
GBE1	-181.4	0.453922659	0

GDE1	-22.75	0.946082766	0
GHITM	430.65	0.064788641	0.012202175
GLOD4	-111.3	0.671396138	0
GNB2L1	582.4	0.018405028	0
GSTP1	637.15	0.013996154	0
GTF2A2	390.15	0.093093614	0.020325203
H2AFZ	583.9	0.01820339	0
H3F3A	570.85	0.020139303	0
HES1	-595.7	0.017080745	0
HIGD1A	517.1	0.028697115	0.002427099
HIST1H4C	557.25	0.022131336	0
HMGB1	682.55	0.013996154	0
HMGB2	443	0.057030693	0.010220625
HMGN2	472.9	0.0420338	0.006729685
HN1	471.7	0.04262297	0.006729685
HNRNPA1	913.45	0.013996154	0
HSBP1	511.85	0.02918997	0.003979921
HSP90AA1	945.55	0.013996154	0
HSP90AB1	596.85	0.017037975	0
HSPA5	-133.45	0.599902575	0
HSPA8	502.65	0.031384181	0.003979921
HSPD1	586.25	0.017979769	0
IDI1	359.2	0.123386114	0.029065631
IL23A	486.65	0.037347545	0.00537723
ILF2	402.1	0.083354037	0.017135771
INHBA	339.4	0.144809524	0.035597457
KIAA0101	886.9	0.013996154	0
KIF20B	-62.7	0.832957675	0
KIF23	474.5	0.041554374	0.006729685
KRT17	-8.6	0.980925751	0
KRT18	645.85	0.013996154	0
KRT8	617.75	0.014809859	0
LAPTM4A	317.05	0.176988327	0.046535053
LCN2	575.2	0.019413613	0
LDHA	545.9	0.022426587	0.002427099
LDHB	558.1	0.02194186	0
LGALS1	-107.85	0.686690011	0
LSM3	323.2	0.166996008	0.043107819
LYST	-25.2	0.939667051	0
MAD2L1	525.35	0.026715254	0.002427099
MAP2K6	-82.65	0.76553128	0
MATR3	534.85	0.024648352	0.002427099
MDH1B	-23.1	0.945102766	0
MED6	529.4	0.025650877	0.002427099
MIF	595.55	0.017080745	0
MIR205HG	-65.25	0.823737794	0
MMADHC	537.05	0.024303371	0.002427099
MMP1	524.45	0.026715254	0.002427099

MORF4L1	-246.6	0.297731827	0
MORF4L2	484.15	0.038124685	0.00537723
MRPL14	553.2	0.022286008	0
MRPL18	331	0.154725823	0.039122829
MT1E	-217.05	0.363047196	0
MT2A	370.1	0.112193904	0.026024952
MTCH2	632.25	0.013996154	0
MTRNR2L2	699.05	0.013996154	0
MTRNR2L8	652.2	0.013996154	0
MTRNR2L9	686.25	0.013996154	0
MYL12A	380.7	0.101242486	0.022339968
MYL6	633.65	0.013996154	0
NAA20	670.8	0.013996154	0
NDUFA11	400.3	0.084490046	0.017135771
NDUFB11	487.6	0.037036458	0.00537723
NDUFB9	560.25	0.021827751	0
NDUFS5	740.05	0.013996154	0
NDUFS6	364.4	0.118913401	0.027243266
NDUFS8	395	0.088857887	0.018754859
NDUFV2	474.6	0.041554374	0.006729685
NEMP1	-6.5	0.985751256	0
NHP2	534.75	0.024648352	0.002427099
NME2	433.85	0.062831439	0.011237424
NOP10	387.85	0.095442529	0.020325203
NPM1	401.2	0.083852761	0.017135771
NQO1	411.2	0.077064967	0.014805306
NTMT1	407.05	0.079128776	0.016476493
NUSAP1	344.75	0.1390783	0.034140469
OLR1	-91.6	0.739489977	0
ORC4	425.65	0.067958484	0.01303284
P2RX4	-35.65	0.914680494	0
PACRGL	-37.45	0.909322742	0
PARK7	354.55	0.127905917	0.030106483
PBK	380.55	0.101242486	0.022339968
PGK1	358.05	0.124601695	0.029539002
PHB	370.6	0.111707412	0.025777964
PKM	608.3	0.015953642	0
PLAU	483.3	0.03829125	0.00537723
PLIN2	391.65	0.091876281	0.019682091
PLRG1	401.45	0.083672068	0.017135771
POLR2L	523.55	0.026770903	0.002427099
POMP	509.1	0.029850592	0.003979921
PPIA	815.45	0.013996154	0
PPIB	460	0.048513187	0.007925753
PRDX1	905.6	0.013996154	0
PRDX3	357.25	0.125726449	0.029539002
PSMA2	645.6	0.013996154	0
PSMA3	513.1	0.028940184	0.003979921

PSMA4	501.35	0.031670391	0.003979921
PSMA6	461.35	0.047711921	0.007925753
PSMA7	523.6	0.026770903	0.002427099
PSMB1	434.7	0.062235238	0.011237424
PSMB4	358.6	0.123964806	0.029065631
PSMB5	460	0.048513187	0.007925753
PSMC3IP	515.75	0.028699686	0.003979921
PSMD10	-14.2	0.965845176	0
PSMD11	551.8	0.022286008	0.002427099
PSMD4	585.2	0.018022857	0
PTHLH	-477.25	0.040501202	0
PTMA	668.2	0.013996154	0
PUM3	378.3	0.103251682	0.023133291
RAB11A	408.25	0.078546549	0.015597162
RABEPK	352.9	0.130115294	0.030625633
RAN	393.55	0.090014032	0.018754859
RBM3	454.6	0.051062633	0.007925753
RBM39	569.7	0.020139303	0
RBM8A	661.8	0.013996154	0
ROMO1	422.05	0.070014965	0.01303284
RPL10	523.8	0.026770903	0.002427099
RPL11	706.25	0.013996154	0
RPL12	449.85	0.05344433	0.009139078
RPL13	452.05	0.052322547	0.009139078
RPL13A	436.3	0.061174664	0.011237424
RPL14	862.65	0.013996154	0
RPL15	658.8	0.013996154	0
RPL18	863.05	0.013996154	0
RPL19	841.4	0.013996154	0
RPL21	606.15	0.015953642	0
RPL22	851.05	0.013996154	0
RPL23	532.75	0.025112727	0.002427099
RPL23A	371.9	0.109960887	0.025324865
RPL24	804.15	0.013996154	0
RPL26	549.75	0.022286008	0.002427099
RPL27A	696.3	0.013996154	0
RPL29	749.3	0.013996154	0
RPL3	512.35	0.029088415	0.003979921
RPL30	684.05	0.013996154	0
RPL31	419.15	0.071359107	0.013991269
RPL32	827.25	0.013996154	0
RPL34	577.45	0.019271739	0
RPL35	480.25	0.039259169	0.00537723
RPL35A	682.95	0.013996154	0
RPL36	749	0.013996154	0
RPL37	1000.45	0.013996154	0
RPL37A	873.2	0.013996154	0
RPL38	490.1	0.035969737	0.00537723

RPL39	314.95	0.178694842	0.047114281
RPL4	574.75	0.019413613	0
RPL41	715.75	0.013996154	0
RPL5	632.8	0.013996154	0
RPL6	549.35	0.022286008	0.002427099
RPL7A	892.6	0.013996154	0
RPL8	732.6	0.013996154	0
RPL9	1028.05	0.013996154	0
RPLP0	576.6	0.019381081	0
RPLP1	740.4	0.013996154	0
RPLP2	1065.35	0.013996154	0
RPS11	565	0.020921569	0
RPS12	806.4	0.013996154	0
RPS13	657.5	0.013996154	0
RPS14	534.55	0.024648352	0.002427099
RPS15	495.25	0.034021739	0.003979921
RPS16	713.85	0.013996154	0
RPS18	556.25	0.022284404	0
RPS19	572.4	0.019857513	0
RPS20	384.45	0.097996494	0.021895171
RPS21	667.8	0.013996154	0
RPS23	635.1	0.013996154	0
RPS24	550.7	0.022286008	0.002427099
RPS26	558.3	0.02194186	0
RPS27A	704.55	0.013996154	0
RPS28	673.55	0.013996154	0
RPS29	820.4	0.013996154	0
RPS3	987.4	0.013996154	0
RPS3A	619.2	0.014803571	0
RPS4X	521.6	0.027092105	0.002427099
RPS5	759.35	0.013996154	0
RPS6	447.55	0.054804481	0.009139078
RPS6KC1	-35.35	0.914684211	0
RPS7	717.9	0.013996154	0
RRP15	433.8	0.062831439	0.011237424
S100A10	419.45	0.071317241	0.013991269
SAA1	359.3	0.123353049	0.029065631
SAT1	378.35	0.103251682	0.023133291
SCEL	530.15	0.025512367	0.002427099
SEC13	587.4	0.017947059	0
SETD4	-48.55	0.877545882	0
SNRPA1	394.95	0.088857887	0.018754859
SNRPB	623.35	0.014630597	0
SNRPD1	605.45	0.015953642	0
SNRPD2	325.6	0.163250252	0.041545501
SNRPE	473.4	0.041881733	0.006729685
SNRPF	539.45	0.023980769	0.002427099
SNRPG	503.3	0.031259943	0.003979921

SOD1	527.15	0.026113793	0.002427099
SOX4	330.7	0.154865128	0.039122829
SPRR1B	-5.5	0.98765097	0
SRP14	516.35	0.028697115	0.003979921
SRSF7	781.3	0.013996154	0
SSB	404.3	0.081905363	0.016476493
STMN1	800.6	0.013996154	0
SUB1	523.45	0.026770903	0.002427099
SUMO2	774	0.013996154	0
TCEAL9	332.35	0.152776915	0.038676348
TCEB2	661.3	0.013996154	0
THOC1	316.3	0.177642166	0.046535053
THRAP3	399.25	0.08524772	0.017891608
THUMPD3	658.15	0.013996154	0
TIMM21	444.25	0.056463	0.010220625
TMA7	-152.1	0.544550977	0
TMBIM6	338.05	0.146326367	0.036694185
TMEM258	636.6	0.013996154	0
TMSB10	657.7	0.013996154	0
TMSB4X	360.9	0.122221879	0.028613746
TOMM22	457.65	0.049574514	0.007925753
TOMM5	376.85	0.104729333	0.023815506
TOP2A	344.45	0.139164621	0.034140469
TP63	-153.2	0.540194337	0
TPI1	583.8	0.01820339	0
TPT1	422.5	0.069832155	0.01303284
TRIT1	-22.2	0.947316632	0
TUBA1B	618.95	0.014803571	0
TUBB	473.95	0.041688967	0.006729685
TUBB4B	842.05	0.013996154	0
TXN	660.7	0.013996154	0
UBA52	570.25	0.020139303	0
UBE2C	549.8	0.022286008	0.002427099
UBE2T	650.3	0.013996154	0
UBL5	499.4	0.032282369	0.003979921
UBXN1	358.5	0.123990303	0.029483606
UQCR10	-93.65	0.730830993	0
UQCR11	485.6	0.037728205	0.00537723
UQCRH	528.1	0.025922145	0.002427099
UQCRQ	316.6	0.177449079	0.046535053
VRK1	644.2	0.013996154	0
WDR54	382.45	0.09951174	0.022228637
XRCC6	515.35	0.028699686	0.003979921
YWHAZ	532.2	0.025142599	0.002427099
ZNF28	-66.3	0.821492442	0
ZNF485	-11.25	0.972718182	0
ZNF77	-10.35	0.975623699	0
ZWINT	546.55	0.022426587	0.002427099

TABLE S4: A total of 91 differentially expressed (DE) genes between cell populations of 5637 vs. stressed 5637.

See below

Table S4. A total of 91 differentially expressed (DE) genes between cell populations of 5637 vs. stressed 5637.

Gene symbol	DE score	*p* value	*q* value
ACTB	-689.55	0.024438596	0
ANXA2	-1071	0.024438596	0
ANXA3	-421.9	0.135391608	0.043868283
ATP5E	-505.55	0.060977987	0.015202218
ATP5F1	-506.55	0.060977987	0.015202218
BANF1	-488.4	0.07223743	0.018788657
BCAP31	-485.9	0.073339674	0.018788657
CAV1	-884	0.024438596	0
CCNB1	-662.65	0.024438596	0
CCNB2	-439.2	0.115655512	0.035396209
CDK1	598.5	0.029905063	0.040982937
CTSV	1008.3	0.024438596	0
DDX5	-590.35	0.031524096	0
EEF1A1	-633.35	0.027564516	0
ENO1	-566.4	0.036525	0
GAPDH	-563.3	0.036834951	0
GNB2L1	-481.7	0.07657672	0.022803327
HAT1	-539.1	0.046813559	0.009881442
HNRNPA1	-1194.9	0.024438596	0
HSP90AB1	-619.95	0.028060606	0
HSPA5	-850.15	0.024438596	0
HSPA8	-706.45	0.024438596	0
KIAA0101	869.85	0.024438596	0
KRT8	-670.9	0.024438596	0
LDHA	-501.3	0.062464072	0.015202218
MIR205HG	-1058.4	0.024438596	0
MNS1	850.4	0.024438596	0
MRPL13	-455.95	0.096980349	0.029644325
MT2A	-801.3	0.024438596	0
NACA	-915.15	0.024438596	0
NPM1	-1042.6	0.024438596	0
PMPCB	-433.8	0.122337165	0.038666511
PPIA	-484.1	0.074610215	0.018788657
PSMA2	-423.15	0.134643617	0.043868283
PSMA3	-417.25	0.142064846	0.045606654
PSMB1	-515.95	0.056430556	0.015202218
PSMB7	-483.7	0.074713904	0.018788657
PTHLH	-466.65	0.089090909	0.026626639
PTTG1	-519.9	0.054278571	0.015202218
RABEPK	-573.1	0.035704301	0
RAN	-586.9	0.032143678	0
RBM3	-899.25	0.024438596	0
RPL10	-655.05	0.024438596	0
RPL10A	-788.5	0.024438596	0
RPL11	-601.9	0.029493421	0

RPL13A	-501.5	0.062464072	0.015202218
RPL14	-537.15	0.047591667	0.009881442
RPL21	-505.6	0.060977987	0.015202218
RPL22	-521.15	0.054134058	0.015202218
RPL24	-593.25	0.030932099	0
RPL26	-784.45	0.024438596	0
RPL3	-925.5	0.024438596	0
RPL30	-448.05	0.104179012	0.032593237
RPL34	-495.55	0.066659884	0.018788657
RPL37	-598.1	0.029905063	0
RPL37A	-656.3	0.024438596	0
RPL4	-782.1	0.024438596	0
RPL41	-480.3	0.077242188	0.022803327
RPL5	-567.6	0.036494898	0
RPL7A	-670.25	0.024438596	0
RPL8	-439.85	0.115543825	0.035396209
RPL9	-588.95	0.031738095	0
RPS11	-486.5	0.073339674	0.018788657
RPS12	-699.2	0.024438596	0
RPS13	-414.9	0.14489094	0.048172028
RPS16	-513.15	0.057789116	0.015202218
RPS21	-556.9	0.038640187	0.009881442
RPS23	-685.1	0.024438596	0
RPS25	-568.25	0.036479381	0
RPS27A	-769.35	0.024438596	0
RPS29	-476.4	0.080281726	0.022803327
RPS3	-674.75	0.024438596	0
RPS3A	-465.5	0.090372038	0.026626639
RPS4X	-517.65	0.055475352	0.015202218
RPS6	-533.7	0.048821138	0.009881442
RPS7	-685.35	0.024438596	0
S100A10	-692.4	0.024438596	0
S100A2	-840.4	0.024438596	0
SAT1	688.35	0.024438596	0.008634269
SEC13	-610.65	0.028676056	0
SEC61G	-510.8	0.059837838	0.015202218
SERF2	-548.85	0.041343478	0.009881442
SNRPE	-537.55	0.047579832	0.009881442
SOX4	885.2	0.024438596	0
SRI	-432.85	0.123617424	0.038666511
SRSF3	-617.2	0.028102941	0
SRSF7	-622.95	0.027792308	0
SUB1	-455.35	0.097504348	0.029644325
TMSB4X	-725.1	0.024438596	0
UBL5	-426.1	0.130938406	0.043868283
XRCC5	-426.2	0.130938406	0.043868283

TABLE S5: A total of 219 overlapped genes differentially expressed (DE) between cell populations of 5637 vs. 5637PR and 5637PR vs. stressed 5637PR.

See below

Table S5. A total of 219 overlapped genes differentially expressed (DE) between cell populations of 5637 vs. 5637PR and 5637PR vs. stressed 5637PR.

Gene symbol	DE score	
	5637 vs 5637PR	5637PR vs Stressed 5637PR
ACTG1	-1350.2	336.5
ADSL	-516	332.75
ARL6IP1	-441.9	-297.65
ATP5A1	-1614	685.9
ATP5L	-1901.55	419.6
ATP6V0B	-1264.5	760.4
AURKA	-728.5	348.9
B2M	-1647.2	439.7
BEX3	-1834.65	385.4
BRIX1	-959.6	657.9
BRK1	-1731.2	361.4
BSG	-882.2	398.5
BTF3	-1804.35	403.9
C11orf58	-1745.8	549.25
C15orf48	-787	490.35
C9orf78	-1190.95	508.95
CACYBP	-1609.45	671.75
CALM2	-1722.2	493.15
CALU	-1784	408.5
CCT2	-1790.4	475.2
CCT3	-1380.1	467.1
CCT5	-1383.75	508.9
CCT7	-1240.6	598.35
CCT8	-1718.35	582.75
CD59	-1529	522.6
CDC5L	-664.3	435.4
CDC6	-997.85	697.2
CDKN3	-1005.05	-45.95
CKS1B	-1180.3	500.55
CLIC1	-1684.9	474.25
CLSPN	-1168.6	888.35
CNIH4	-1564.9	312.25
COPS8	-1148.05	353.15
COX4I1	-1741.9	579.85
COX7B	-1961.35	-36.4
COX8A	-1711.75	515.6
CSF2	-304.85	586.05
CSTB	-1182.1	334.4
CTSC	-1426.75	605.8
DNAJA1	-1560.8	904.95
DPY30	-1249.5	449.2
DYNLL1	-1739.05	455.1
DYNLT1	-1469.65	316

EBNA1BP2	-1327.25	548.95
EDF1	-1377.25	630.5
EED	-686.5	360
EEF1B2	-1791.1	685.6
EEF1D	-1503.75	555.9
EEF1G	-1805.1	536.05
EIF1	-1749.65	795.65
EIF2S1	-1590.45	579.4
EIF3E	-1184.65	-28.35
EIF3I	-1826.4	607.05
EIF3M	-1785.8	379.9
EIF4A2	-1116.55	337.95
EIF4E2	-1370.4	383.8
EMC3	-1563.7	546.45
ESCO2	-678.95	656.45
FAM96A	-1239	437.85
FANCD2	-422.5	412.85
FANCI	-514.2	327.25
FAU	-1855.65	822.1
FTH1	-748.4	429.95
FTL	-2009.05	915.4
GHITM	-1425.55	430.65
GSTP1	-1901.95	637.15
GTF2A2	-1385.7	390.15
H2AFZ	-1906.35	583.9
H3F3A	-1475.25	570.85
HES1	-839.15	-595.7
HIGD1A	-1701.95	517.1
HIST1H4C	-1506.95	557.25
HMGB1	-1959.05	682.55
HMGB2	-1131.7	443
HMGN2	-1865.55	472.9
HN1	-1394.55	471.7
HSBP1	-1515.4	511.85
HSP90AA1	-1964.6	945.55
HSPD1	-1883.95	586.25
IDI1	-708.25	359.2
ILF2	-1624.85	402.1
INHBA	-1781.05	339.4
KIF23	-364.4	474.5
KRT17	-431.25	-8.6
KRT18	-1186.9	645.85
LAPTM4A	-1369.85	317.05
LDHB	-1796.35	558.1
LGALS1	-837.75	-107.85
LSM3	-1616.85	323.2
MAD2L1	-1079.5	525.35
MATR3	-1256.85	534.85

MED6	-685.5	529.4
MIF	-769.65	595.55
MMP1	-318.45	524.45
MORF4L1	-857.7	-246.6
MORF4L2	-1658.7	484.15
MRPL14	-910.35	553.2
MRPL18	-1309.05	331
MT1E	-430.3	-217.05
MTCH2	-1465.05	632.25
MTRNR2L2	-1972.2	699.05
MTRNR2L8	-1970.75	652.2
MTRNR2L9	-1607.95	686.25
MYL12A	-1655.6	380.7
MYL6	-1970.2	633.65
NAA20	-1500.3	670.8
NDUFA11	-1371.7	400.3
NDUFB11	-1342.55	487.6
NDUFB9	-1779.05	560.25
NDUFS5	-1924.3	740.05
NDUFS6	-693.35	364.4
NDUFS8	-1350.3	395
NDUFV2	-1433.45	474.6
NHP2	-1469.4	534.75
NME2	-1665.55	433.85
NOP10	-1857.45	387.85
NQO1	-1109.3	411.2
NTMT1	-983.75	407.05
NUSAP1	-995.5	344.75
PARK7	-1731.5	354.55
PBK	-562.65	380.55
PGK1	-1671.65	358.05
PHB	-1636.9	370.6
PKM	-1230.1	608.3
PLAU	-528.95	483.3
PLIN2	-598.95	391.65
PLRG1	-637.45	401.45
POLR2L	-1757.65	523.55
POMP	-1962.7	509.1
PPIB	-1294.4	460
PRDX1	-1931.7	905.6
PRDX3	-924.25	357.25
PSMA4	-1778.8	501.35
PSMA6	-1717.6	461.35
PSMA7	-1546.15	523.6
PSMB4	-1266.75	358.6
PSMB5	-1711.95	460
PSMC3IP	-659.45	515.75
PSMD10	-1064.8	-14.2

PSMD11	-1595.35	551.8
PSMD4	-1248.75	585.2
PTMA	-2002.5	668.2
PUM3	-1514.9	378.3
RAB11A	-758.95	408.25
RBM39	-1713.25	569.7
RBM8A	-1812.65	661.8
ROMO1	-1765.55	422.05
RPL12	-1824.75	449.85
RPL13	-796.9	452.05
RPL15	-1930.2	658.8
RPL18	-1917.8	863.05
RPL19	-1965.15	841.4
RPL23	-1923.1	532.75
RPL23A	-2006.7	371.9
RPL27A	-1860.9	696.3
RPL29	-1827.85	749.3
RPL31	-1982.55	419.15
RPL32	-2026.1	827.25
RPL35	-2011.8	480.25
RPL35A	-1921.25	682.95
RPL36	-1980.3	749
RPL38	-1996.5	490.1
RPL39	-2023.3	314.95
RPL6	-1932.35	549.35
RPLP0	-1785.15	576.6
RPLP1	-1940.9	740.4
RPLP2	-1988.1	1065.35
RPS14	-1926.7	534.55
RPS15	-1604.35	495.25
RPS18	-1965.25	556.25
RPS19	-2041.55	572.4
RPS20	-1689.35	384.45
RPS24	-1970.4	550.7
RPS26	-2032.7	558.3
RPS28	-1962	673.55
RPS5	-1935.95	759.35
RRP15	-1103.5	433.8
SNRPA1	-1215.6	394.95
SNRPB	-716.75	623.35
SNRPD1	-1723.85	605.45
SNRPD2	-2033.35	325.6
SNRPF	-1892.5	539.45
SNRPG	-1973.35	503.3
SOD1	-1711	527.15
SRP14	-1960.7	516.35
SSB	-1635.75	404.3
STMN1	-1757.2	800.6

SUMO2	-1859.15	774
TCEAL9	-1702.15	332.35
TCEB2	-1628.55	661.3
THOC1	-232.5	316.3
THRAP3	-1273.7	399.25
THUMPD3	-1581.6	658.15
TIMM21	-865.45	444.25
TMA7	-1884	-152.1
TMBIM6	-1823.45	338.05
TMEM258	-1884.3	636.6
TMSB10	-1978.3	657.7
TOMM22	-1044.25	457.65
TOMM5	-1850.3	376.85
TOP2A	-924.55	344.45
TPI1	-1833.7	583.8
TPT1	-1898.4	422.5
TUBA1B	-2010.9	618.95
TUBB	-1360.3	473.95
TUBB4B	-1067.75	842.05
TXN	-2027.95	660.7
UBA52	-2028.3	570.25
UBE2C	-561.5	549.8
UBE2T	-941.2	650.3
UBXN1	-512.45	358.5
UQCR10	-1546.5	-93.65
UQCR11	-1481.25	485.6
UQCRH	-1938.65	528.1
UQCRQ	-1857.55	316.6
VRK1	-668.25	644.2
XRCC6	-1025.95	515.35
YWHAZ	-1443.95	532.2
ZWINT	-701.5	546.55

				Pre-NAC (Dx)		Post-NAC (Cystectomy)			
Pt	Gender	Age at cystectomy	NAC	Tumor grade	Histology	Pathological T stage	Tumor grade	Histology	LNI
1	Male	57	MVAC	HG	UC	2	HG	UC	No
2	Male	61	MVAC	HG	UC	3	HG	UC	No
3	Male	44	MVAC	HG	UC, AC	3	HG	UC	No
4	Male	65	GC, 5FU	HG	UC, AC	3	HG	UC, AC	Yes
5	Male	62	GC, MVAC	HG	UC	3	HG	UC, AC	Yes
6	Male	57	GC	HG	UC	3	HG	UC	Yes
7	Female	75	GC	HG	UC	3	HG	UC	No
8	Male	77	GC	HG	UC	2	HG	UC	No
9	Female	80	TIP	HG	UC, SCC	3	HG	UC	Yes
10	Male	65	GC	HG	UC	3	HG	UC	Yes
11	Male	77	GC	HG	UC	2	HG	UC	No
12	Male	70	GC	HG	UC	4	HG	UC	Yes
13	Female	70	GC	HG	UC	1	HG	UC	No
14	Male	79	GC	HG	UC	4	HG	UC	No
15	Male	69	GC	HG	UC, AC, SCC	a	HG	UC	Yes
16	Female	47	MVAC	HG	UC	2	HG	UC, SCC	No
17	Female	73	-	LG	UC, SCC	1	HG	UC, SCC	No
18	Male	70	-	HG	UC, SCC	4	HG	UC, SCC	No
19	Female	76	-	HG	UC	3	HG	UC	No
20	Female	74	-	HG	UC	2	HG	UC	No
21	Male	74	-	HG	UC	1	HG	UC	No
22	Male	63	-	HG	UC, AC	2	HG	UC, AC	Yes
23	Female	34	-	HG	UC	3	HG	UC, Sarcomatoid	Yes
24	Male	75	-	HG	UC	3	HG	UC	No
25	Male	74	-	HG	UC	4	HG	UC	No
26	Male	77	-	HG	UC	1	HG	UC	No

TABLE S6: Summary of the clinical information for 26 patients with urinary bladder cancer, with (n = 16) and without (n= 10) neoadjuvant CDDP-based chemotherapy (NAC). Dx, diagnosis; LNI, lymph node involvement; MVAC, methotrexate, vinblastine, doxorubicin and CDDP; GC, gemcitabine, CDDP; 5FU, 5-fluorouracil; TIP, Paclitaxel, ifosfamide, and CDDP; HG, high grade; UC, urothelial carcinoma; AC, adenocarcinoma; SCC, squamous cell carcinoma.

Name	Sequence (5'->3')
si- *PSMD10*	Sense: GCUGUACUCCCUUACAUUAtt<br>Antisense: UAAUGUAAGGGAGUACAGCca
si- *CDKN3*	Sense: CCAUCAAGCAAUACAAUUAtt<br>Antisense: UAAUUGUAUUGCUUGAUGGtc
si- *MORF4L1*	Sense: ACAGUAUGCUGAAAUUCUUtt<br>Antisense: AAGAAUUUCAGCAUACUGUgg
si- *UQCR10*	Sense: AGAUAUUACAGUCAACUUUtt<br>Antisense: AAAGUUGACUGUAAUAUCUcc
si- *HES1*	Sense: GCCUAUUAUGGAGAAAAGAtt<br>Antisense: UCUUUUCUCCAUAAUAGGCtt
si- *ARL6IP1*	Sense: GGACUAAACCAACAUGGAAtt<br>Antisense: UUCCAUGUUGGUUUAGUCCag
si- *TMA7*	Sense: CCAUAACUGUGAAUUUAAAtt<br>Antisense: UUUAAAUUCACAGUUAUGGta
si- *EIF3E*	Sense: GGAAGACCUUACACGGUUAtt<br>Antisense: UAACCGUGUAAGGUCUUCCat
si- *KRT17*	Sense: CCGUCAGGUGCGUACCAUUtt<br>Antisense: AAUGGUACGCACCUGACGGgt
si- *LGALS1*	Sense: GAUGGAUACGAAUUCAAGUtt<br>Antisense: ACUUGAAUUCGUAUCCAUCtg
si- *MT1E* (A)	Sense: GUAAAUAGAACAACCUGCAtt<br>Antisense: UGCAGGUUGUUCUAUUUACat
si- *MT1E* (B)	Sense: AAUACAACACUGAGCCAUUtt<br>Antisense: AAUGGCUCAGUGUUGUAUUtt
si- *COX7B* (A)	Sense: AAGUCGGAAUAGAAUGGAAtt<br>Antisense: UUCCAUUCUAUUCCGACUUgt
si- *COX7B* (B)	Sense: CUGAUUUUCAUGACAAAUAtt<br>Antisense: UAUUUGUCAUGAAAAUCAGgt

TABLE S7: List of siRNAs used in the present study.
